# Transmission modeling to infer tuberculosis incidence prevalence and mortality in settings with generalized HIV epidemics

**DOI:** 10.1038/s41467-023-37314-1

**Published:** 2023-03-24

**Authors:** Peter J. Dodd, Debebe Shaweno, Chu-Chang Ku, Philippe Glaziou, Carel Pretorius, Richard J. Hayes, Peter MacPherson, Ted Cohen, Helen Ayles

**Affiliations:** 1 School of Health and Related Research, University of Sheffield, Sheffield, UK; 2 School of Public Health, Infectious Disease Epidemiology, Imperial College London, London, UK; 3 Global TB Programme, World Health Organization, Geneva, Switzerland; 4 Avenir Health, Glastonbury, CT USA; 5 Department of Infectious Disease Epidemiology, Faculty of Epidemiology and Population Health, London School of Hygiene and Tropical Medicine, London, UK; 6 School of Health & Wellbeing, University of Glasgow, Glasgow, UK; 7 Clinical Research Department, Faculty of Infectious and Tropical Diseases, London School of Hygiene and Tropical Medicine, London, UK; 8 Department of Epidemiology of Microbial Diseases, Yale School of Public Health, New Haven, CT USA; 9 ZAMBART Project, Ridgeway Campus, University of Zambia, Lusaka, Zambia

**Keywords:** Tuberculosis, HIV infections, Epidemiology, Computational models

## Abstract

Tuberculosis (TB) killed more people globally than any other single pathogen over the past decade. Where surveillance is weak, estimating TB burden estimates uses modeling. In many African countries, increases in HIV prevalence and antiretroviral therapy have driven dynamic TB epidemics, complicating estimation of burden, trends, and potential intervention impact. We therefore develop a novel age-structured TB transmission model incorporating evolving demographic, HIV and antiretroviral therapy effects, and calibrate to TB prevalence and notification data from 12 African countries. We use Bayesian methods to include uncertainty for all TB model parameters, and estimate age-specific annual risks of TB infection, finding up to 16.0%/year in adults, and the proportion of TB incidence from recent (re)infection, finding a mean across countries of 34%. Rapid reduction of the unacceptably high burden of TB in high HIV prevalence settings will require interventions addressing progression as well as transmission.

## Abstract (web summary)

Accurately estimating the burden of tuberculosis is challenging due to incomplete registration systems and the relationship with HIV. Here, the authors develop a Bayesian modelling strategy accounting for these factors that estimates age- and country-specific annual risks of infection and the proportion resulting from recent infection.

## Introduction

Over the past decade, tuberculosis (TB) has killed more people than any other pathogen, averaging more than 1.65 million deaths per year between 2010-2019, of which > 363,000 deaths per year were among people living with HIV (PLHIV)^1^. HIV infection has a large impact on TB in individuals: risks of developing TB increase steeply as CD4 cell counts decline, as does the likelihood of death among those not receiving treatment for TB^2^. Antiretroviral therapy (ART) for PLHIV increases life-expectancy^3^, and reduces TB mortality^4^ and incidence^5^ (although not to the level of HIV-uninfected people)^6^. This has led to dynamic TB epidemics in settings with generalized HIV epidemics, with rapid increases in TB notifications driven by declines in mean CD4 cell counts among people living with HIV (PLHIV), followed by declines in TB notifications as ART coverage has increased^7^. Globally in 2020, 8% of TB incidence and 14% of all TB deaths occurred among PLHIV^1^. In the World Health Organization (WHO) African region, these figures are 24% and 31%, respectively; 22 out of the 30 WHO high TB/HIV countries are in Africa^1^.

Our understanding of the global epidemiology of TB is based on estimates of disease incidence and mortality, notably those generated annually by WHO. In many countries with high TB incidence, the gap between estimated TB incidence and cases detected and reported as starting anti-TB treatment (notifications) is substantial, complicating surveillance and accurate measurement of epidemic trajectories. WHO has supported nationally-representative TB prevalence surveys in priority countries, as these provide a measure of TB burden that is less subject to bias than notification data^8–10^. Since 1990, 43 national TB prevalence surveys have been undertaken and reported results^1^, including 16 in Africa^9^. The large sample sizes required mean TB prevalence surveys are expensive to execute, and they are typically powered to generate a relative precision of only + /− 20%^10^.

Combining data from prevalence surveys with notifications data (and potentially vital registration data) to produce estimates of incidence and mortality in high HIV-prevalence settings requires a framework that can incorporate the evolving relationship between incidence, prevalence, and mortality, the effects of HIV, and which can formally account for the uncertainty and relative informativeness inherent in each source of data. Current approaches to TB/HIV burden estimation use dichotomized states to represent HIV and ART which do not capture their evolving and context-specific relationship with TB risk.

Models of TB transmission calibrated to empirical data provide a potential alternative approach to estimating global, regional and national TB burden, but have not been used to date due to technical challenges including the complexity of TB models (especially TB/HIV models), the large number of uncertain parameters, and limitations in surveillance data^11^. Due to slow timescales in TB progression, models need to be run over timescales representing decades, and evolving age-structures are likely to be important determinants of TB trends^12^. HIV is strongly patterned by age and sex, and evolving population CD4 cell count distributions among PLHIV and increasing ART coverage result in a dynamic relationship between HIV and TB increasing the model complexity. Many of the parameters describing the natural history of TB are imprecisely known due to long timescales, rareness of measurable outcomes, and suboptimal diagnostic accuracy.

Calibrating transmission models to data also provides a way to estimate important, transmission-specific epidemiological metrics that are difficult or impossible to measure directly. Patterns by age of infection risk or contribution to the force-of-infection can identify age groups for efficiently targeted interventions to reduce either exposure risks or prevalent disease that is driving transmission. For example, if in a particular country working-age, people contributed a disproportionately large force-of-infection, then a public health strategy of targeted community screening for this group might be an efficient approach to rapidly reduce the incidence of infection—and hence disease—for adults and children. The proportion of TB incidence due to infection or re-infection within a given period is important for projecting trends, and because it quantifies the extent to which interventions can generate indirect benefits through reduced TB incidence, it influences the best choice or design of intervention^13^.

Here we present a novel transmission model/calibration framework that incorporates the dynamic complexity of HIV-fueled TB epidemics and demographic change. We are able to calibrate this model to surveillance data in a likelihood-based formal Bayesian approach with all parameters considered as uncertain. In applying this framework to the 12 WHO high TB/HIV countries in Africa with a nationally representative TB prevalence survey, we provide a new approach to estimating TB burden over time in these settings, and estimate new age-specific metrics of transmission.

## Results

**Fig. 1:**
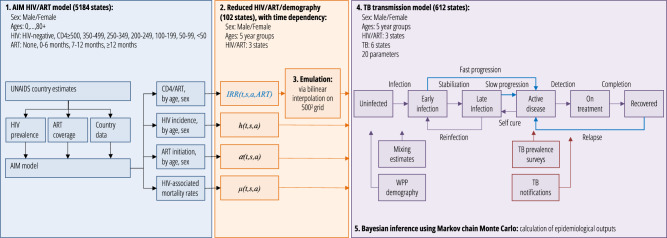
Process flow and model diagram. Blue lines for the TB transmission model in step 4 are processes to which HIV/ART-associated incidence rate ratios *IRR(t,s,a,ART)* are applied. These time-dependent IRRs (step 2) capture the implications of the AIM model of HIV/ART progression (step 1) for TB risk for each aggregated age, sex, and HIV/ART stratum in the simplified transmission model (step 4). The influence of ART-protection and CD4 risk dependence parameters on simplified transmission model dynamics (step 4) is achieved by emulating the dependence of IRR trajectories on these model parameters (step 3), allowing them to be included in inference (step 5). Red boxes in step 4 represent calibration targets.

Below, we present fits of our simplified TB transmission model (see Fig. 1) to available demographic and epidemiological data, and our inferred estimates of national-level TB incidence and mortality, the proportion of TB incidence in PLHIV, the proportion of TB incidence due to infection within the last two years, and also TB infection risks and age-specific contributions to transmission.

### Fits to evolving demography and HIV epidemics

**Fig. 2:**
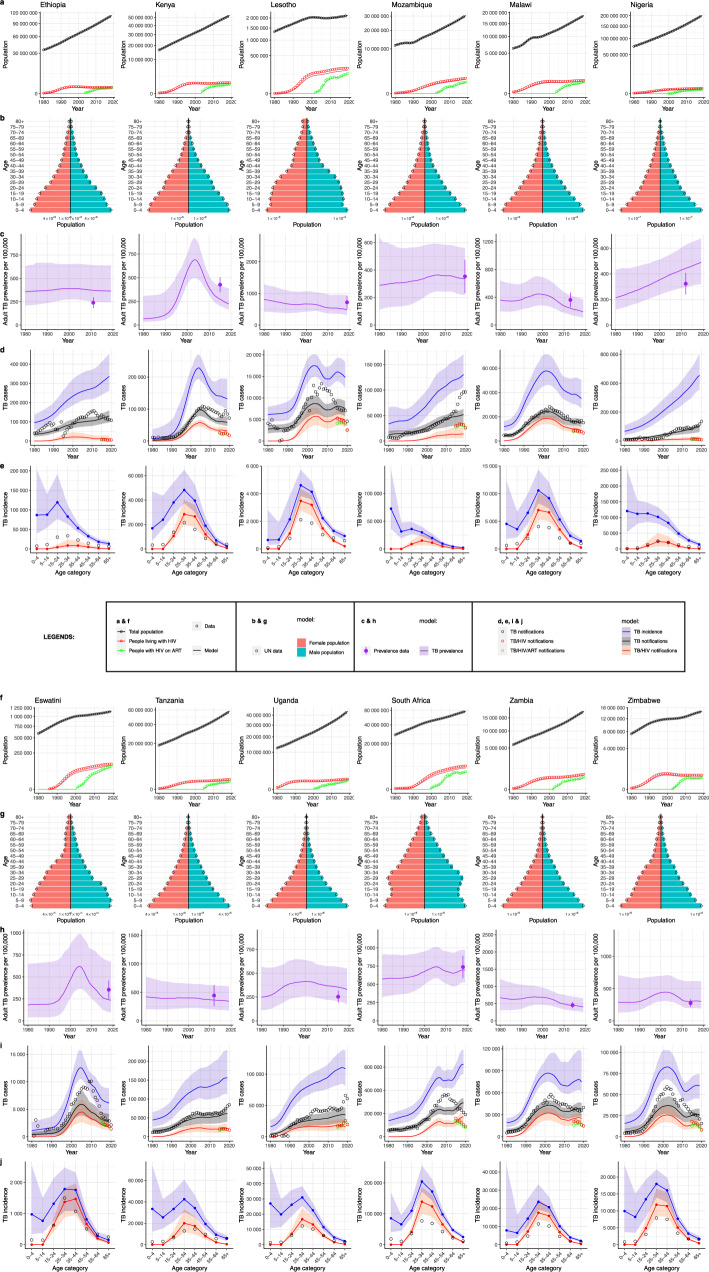
Model outputs compared to empirical data for 12 African countries. Rows **a**, **f** total population (black), people living with HIV (red), people on ART (green) 1980–2019 - points=data, lines=model. Rows **b**, **g**: demographic snapshot in 2015 red/left=women, blue/right=men, points=data. Rows **c**, **h**: per capita TB prevalence 1980–2019, (line = median, ribbon = 95% credible interval [CrI]), and TB prevalence survey data (point=central estimate; error bar = 95% confidence interval). Rows **d**, **i**: incident TB 1980–2019, lines = median/ribbon = 95%CrI for model, points=data, blue = TB incidence, black = notified TB, red = notified TB/HIV, green=notified TB/HIV on ART (data). Rows **e**, **j**: incident TB in 2015 by age, lines = median/ribbon = 95%CrI for model, points = data, blue = TB incidence, black = notified TB, red = incident TB/HIV. All medians and CrIs are based runs using *n* = 300 random samples from the posterior parameter distribution for each country.

Our framework accurately captured changes in national population size and age-sex structure between 1980 and 2019, as well as the number of PLHIV and the number receiving ART (Fig. 2, rows a and b). As expected, we found accelerated increases in the numbers of PLHIV across countries commencing between 1980 and 1990, and rapid increases in ART coverage beginning in the early 2000s, rising to near universal coverage in most countries by 2020.

To make inference tractable, we used a five-year age and sex-structured TB transmission model with HIV/ART (Fig. 1: all states either HIV-negative, HIV-infected/off-ART, HIV-infected/on-ART, giving a total of 612 states). To capture the dynamics of population immunocompetence, model inputs included results from a modified version of the AIM HIV model (which includes detailed CD4 states and progression, totalling 5184 states) on the time-dependent incidence rate-ratios (IRRs) for incident TB relative to HIV-uninfected, and HIV-related mortality, specific to each age and sex state^14,15^. Because the parameters governing the link between IRR and CD4 cell-count and describing the protection from ART against TB were included in our inference, we used a bilinear approximation to accurately emulate the parameter dependence of these inputs (see Supplementary Information for results on approximation).

### Fits to TB data

**Fig. 3:**
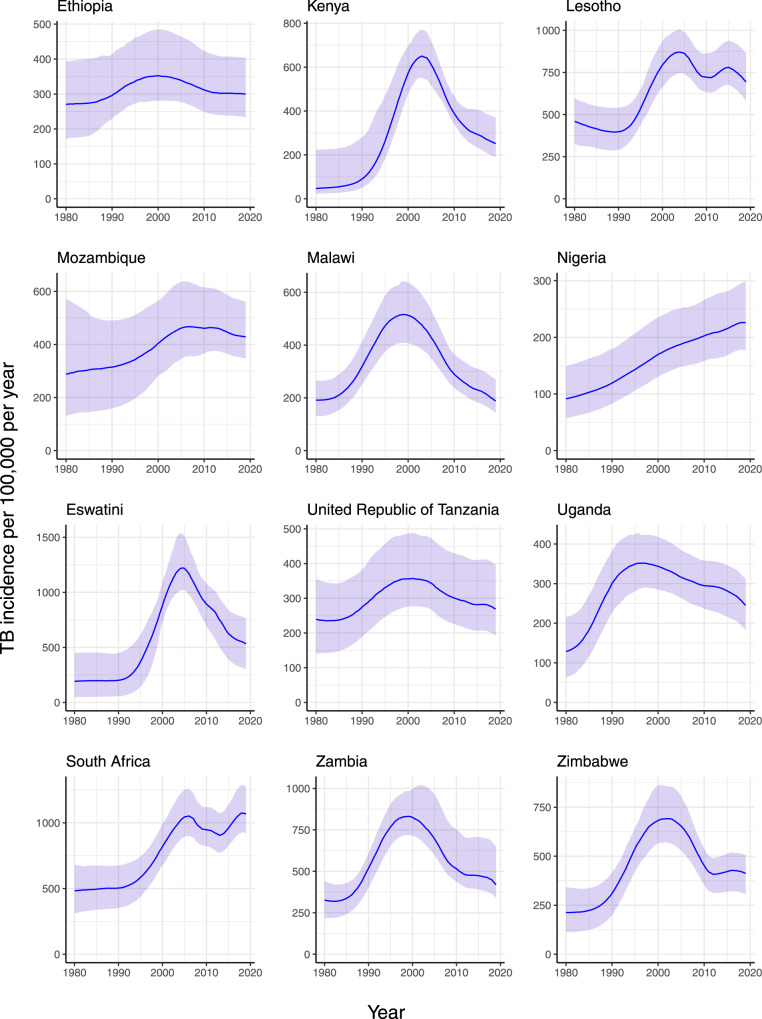
Per capita TB incidence estimated for 12 African countries, 1980–2019. Line = median, ribbon = 95% credible interval [CrI]. All medians and CrIs are based runs using *n* = 300 random samples from the posterior parameter distribution for each country.

TB incidence estimates (Fig. 2, row d) were increasing in all countries in the year 2000 due to a combination of population growth and HIV, with some countries experiencing dramatic accelerations. Per capita incidence has decreased from a peak between 2000 and 2010 in all countries except South Africa and Nigeria (see Fig. 3), but remains higher in absolute numbers in 2019 than in 2010 for 6 out of 12 countries.

We estimate the TB case detection ratio (the ratio of notifications to incidence in a year) now ranges from 31% (95%UI: 24–40%) in Nigeria to 89% (95%UI: 68–109%) in Mozambique, with a median over country central estimates of 58%. Case detection was estimated to be lower in younger children. The uncertainty in case detection reflects the weakly informative prior used for the baseline detection probability and the level of noise inferred from the notification data by the hierarchical model.

TB incidence was highest among 25–34-year-olds for 9 of the 12 countries (Fig. 2, row e), a notable exception being Mozambique where age-stratified notifications were absent.

Uncertainty in our estimates of TB-related metrics (Fig. 2, rows c, d, and e) reflected the limited precision of the data and the 20 uncertain parameters around the natural history of TB and case detection, and their interactions with HIV, which were estimated in our Markov chain Monte Carlo (MCMC) inference.

Effective sample sizes (ESS) obtained using an ensemble slice sampling MCMC approach ranged from 393 to 909, with a mean of 605. Priors, posteriors, and bivariate marginal density plots are presented for each country in the Supplementary Information. Judged by means across countries, the strongest five posterior parameter correlations were between: case detection baseline and trend (*cor(K,c)* *=* *−0.54*); fast progression and transmission (*cor(ε,β)* *=* *−0.53*)*;* fast progression and IRR CD4-dependence (*cor(ε,α)* *=* *−0.50*); progression and relative detection in under 5-year-olds (*cor(*${\varepsilon }_{04}$,${{OR}}_{04}$*)* *=* *−0.33*); and case detection trend and HIV-negative untreated TB duration (*cor(c,D*^*X*^*)* = + *0.30*).

The relative precision (SD/mean) of national adult TB prevalence estimates (children aged < 15 years are not included in TB prevalence surveys) included in the data likelihood ranged from SD/mean = 9% to SD/mean = 23% with a median of 13%; the relative precision of our prevalence estimates in years corresponding to prevalence surveys ranged from 16% to 62% with a median of 27%, but was more uncertain in 1980 (Fig. 2, rows c). Most countries had estimated trends in prevalence that are currently flat or declining, with the exception of Nigeria.

### Comparison with other estimates

**Fig. 4:**
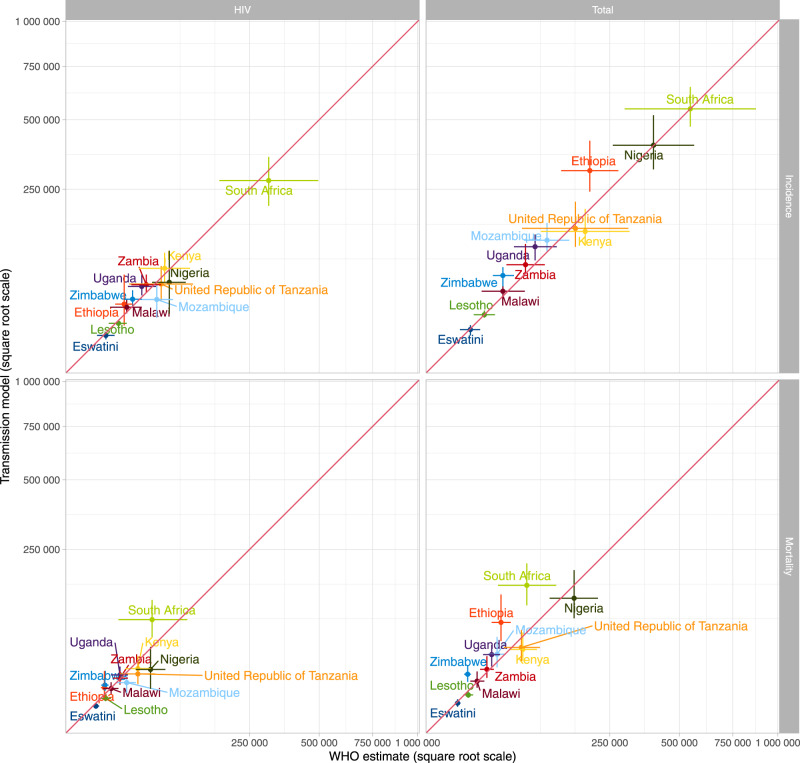
Comparison with WHO incidence and mortality estimates for all TB and TB/HIV for 2019. Line=central estimate, error bar = 95% uncertainty interval [UI]. All model central estimates (defined as medians) and UIs (defined as 95% credible intervals) are based runs using *n* = 300 random samples from the posterior parameter distribution for each country. Central estimates and UIs for WHO data (x-axis) are as reported.

There are no data on mortality for direct comparison: among our 12 countries of interest, only South Africa has a vital registration system and substantial coding of HIV deaths as TB means comparison with raw counts is inappropriate. However, our estimates for 2019 were comparable with WHO estimates (Fig. 4).

### Epidemiological patterns

**Fig. 5:**
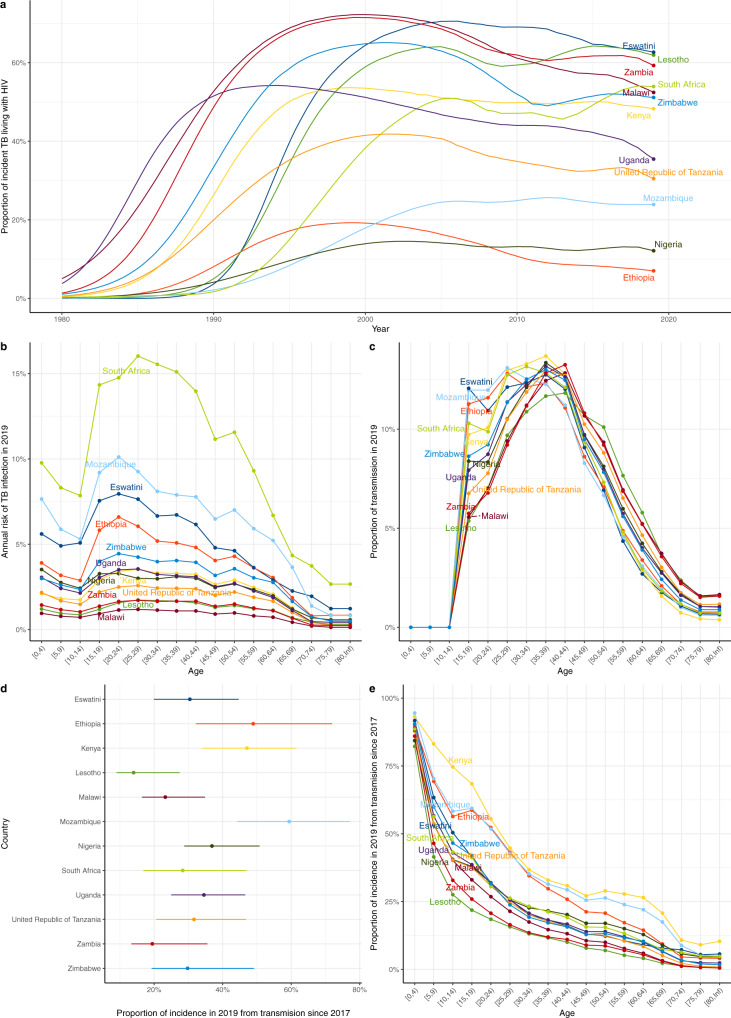
Estimates of other epidemiological metrics. **a** The proportion of incident TB that is TB/HIV for 1980–2019. **b** Annual risk of TB infection in 2019 by age. **c** Proportion of TB transmission from each age group in 2019. **d** Proportion of all TB incidence in 2019 due to (re)infection within 2 years (error bar = 95% credible interval [CrI]). **e** Proportion of all TB incidence in 2019 due to (re)infection within 2 years for each age group. All points/lines=medians. All medians and CrIs are based runs using *n* = 300 random samples from the posterior parameter distribution for each country.

Our estimates show how the proportion of TB incidence that is HIV-associated has evolved over time (Fig. 5a), reducing in 9/12 countries since 2000, when the mean proportion across countries was 47% to a mean of 41% in 2019. However, this proportion remained high in many countries in 2019, ranging from 7% (95%UI: 2–17%) in Ethiopia to 63% (95%UI: 45–76%) in Eswatini, despite very high ART coverage.

We estimate a high and age-dependent annual risk of TB infection (ARTI), reflecting age-assortative mixing and age patterns of infectious TB (Fig. 5b). The ARTI rose through adolescence typically peaking at 20–30 years of age, with this peak ranging from 1.2%/year (95%UI: 0.7%/year to 2.7%/year) in Malawi to 16.0%/year (95%UI: 7.7%/year to 36.1%/year) in South Africa among 25–29 year olds. Of all sources of TB transmission, we estimate that the highest contribution was from people in the 35–39 year age group, with those aged 25–44 years together accounting for between 44% and 53% of transmission across countries (Fig. 5c). We found that the mean proportion of TB incidence due to recent infection (within the last two years) across countries was 34% in 2019, and varied from 14% (95%UI: 9–27%) in Lesotho to 59% (95%UI: 44–77%) in Mozambique (Fig. 5d). This varied strongly by age, decreasing from a mean over countries of 89% of incidence in those aged 0–4 years to < 5% of incidence in those aged over 70 years. Sensitivity analyses on the proportion of TB that is extrapulmonary and assuming children 10–14 years were 50% as infectious as adults rather than uninfectious resulted in median relative changes in central estimates across countries of < 4%. Assuming children 10–14 years were infectious implied they generated a median across countries of 5% of TB transmission (range: 3–9%).

## Discussion

By calibrating a transmission model of TB on an evolving background of demography and HIV-related immunocompetence, we have been able to reproduce patterns in empirical TB data and provide a new method for estimating TB burden in settings with TB prevalence surveys and generalized HIV epidemics. Previous approaches to modelling national-level TB epidemics have had to use existing burden estimates as calibration targets and severely limit the number of model parameters treated as uncertain. Our key innovation is to fit an age-structured TB transmission model to empirical data in a fully-Bayesian framework, generating independent epidemiological and burden estimates while reflecting parametric uncertainty. Important new findings arising from this approach include elucidation of strongly age-dependent proportions of TB incidence from infection within the last two years, and that while TB incidence and mortality have declined in the ART era, TB incidence remains enduringly high with nearly half of TB incidence in these countries among people living with HIV, despite rapid scale-up of ART. These findings suggest that, as ART programmes reach high coverage in many African countries, renewed focus on tuberculosis detection, care and prevention in PLHIV (as well as among HIV-negative people) will be required to accelerate progress towards elimination. Potential approaches include expansion of regular, routine TB screening for people attending HIV care clinics (e.g., annual chest X-ray screening), implementation and expansion of shorter, more tolerable TB preventive therapies, and action to tackle the social determinants and consequences of TB.

Our approach advances the methods used by WHO and IHME for TB burden estimation^16,17^. In settings with TB prevalence surveys but lacking reliable vital registration data, WHO uses estimates of TB duration and CDR estimates from expert opinion and surveillance system review to estimate incidence and mortality^16^. IHME estimates are based on a multi-cause regression approach to predicting mortality^18^, combined with a model of case-fatality and duration (DisMod)^19^ to utilize TB prevalence survey data. Our approach explicitly includes the processes giving rise to TB incidence, prevalence and mortality, includes prior information from our understanding of natural history, and makes only weak prior assumptions about case detection. The level of case detection is determined by the shape of the epidemic and observed prevalence. While our focus here has been on settings where HIV is a key driver of TB, this framework could be easily adapted to lower HIV prevalence settings with TB prevalence surveys. The framework could incorporate vital registration data where available, as well as data not currently used in TB burden estimation, such as TB infection surveys. Application to other settings could quantify the value of the information provided by prevalence surveys in terms of more accurate model-based estimates of burden.

TB transmission models at a country level are mainly used to project the impact of interventions, plan health services, and undertake economic evaluations. Probabilistic sensitivity analyses are well-recognized in health economics as an important component of analyses, not just to adequately convey uncertainty to decision-makers, but because accurately quantifying uncertainty may influence mean results and therefore the outcome of applying a decision rule^20^. Fully quantifying uncertainty is more difficult in the case of transmission models because calibration is required to generate parameter samples for analyses. While our emphasis here has been on burden estimation, for which fully capturing uncertainty is also recognized as important^21^, our technical innovations allowing full characterization of uncertainty based on empirical data would allow economic evaluations of TB interventions to improve their quantification of uncertainty.

Typical practice in calibrating country-level TB transmission models reflects the technical challenges of inference with weak data of different types, and the complex, slow, and uncertain natural history of TB, implying many parameters with uncertain priors. Overcoming these challenges has required particular innovations. The first is developing an approach to represent notification and prevalence data in the likelihood which appropriately weights each data source without ad hoc assumptions. We allowed for a natural noise level in the notification data, and by integrating out this parameter before inference, we were able to avoid introducing an additional parameter to be sampled. The second is the application of state-of-the-art self-tuning MCMC approaches in order to achieve adequate inference for the 20 parameters in our model. We experimented with a number of tools, mainly developed for cosmological model fitting, and here present results based on the Zeus implementation of ensemble slice sampling^22^. Thirdly, in order to maintain the computational efficiency needed for inference while capturing the full complexity of population immuno-dynamics implied by the AIM model, we used emulation (metamodelling) to capture the changing impact of HIV on TB risk over time, and by age and sex, in our simpler transmission model.

Our work nevertheless has limitations. One important limitation is that while we have included sex strata in our model, we have not modelled sex-specific differences in exposure, natural history, and detection, and therefore not made use of sex-strata in data. In most settings, men have more TB than women, and evidence of poorer care access^23^. The extent to which this is driven by different exposure, sex-assortative mixing patterns, differences in risk factors or biology, and differences in care seeking is not fully elucidated^24^. Including sex differences would require several additional uncertain parameters and sex-stratified data to inform them. We have also not included factors other than HIV/ART that may differ between populations and affect TB natural history, nor did we explicitly include the effects of TB preventive therapy (which remains at low levels in these countries). Levels of adherence to ART or anti-TB treatment were not modelled, but could represent the focus of future explorations.

Model TB notifications do not fit through the entire history of notification data for all countries. This partly reflects the limited ability of a two-parameter model of case detection to capture any changes over a 40-year period, and in some countries (eg South Africa), implied upticks in TB incidence due to our version of the AIM model slowing down ART initiations so as not to exceed ART coverage data. A more flexible model of TB detection able to incorporate shocks and periods of worsening service - as well as improvements - would be valuable (see for example the notification trend in Ethiopia), but would need substantially more parameters to be inferred, and systematic collation of comparable (between country) health service data over time. In order to maintain consistency with official UNAIDS HIV/ART estimates that benefit from country data and experience we fitted to HIV/ART modelled estimates, and because HIV/ART estimates are more precise, we neglected their uncertainty in our TB estimation process. This means we have likely underestimated uncertainty, especially in relation to TB/HIV, and are unsure whether there is evidence for ART initiation slow-downs in countries like South Africa, Lesotho, and Zambia. However, this effect highlights the potential for disruptions to HIV/ART services to result in spikes in TB incidence.

We modelled the gap between incidence and notifications as being due to underdiagnosis, and so did not consider overdiagnosis of TB, or undernotification of diagnosed TB. These effects are hard to quantify, but may be important when applying this approach to other settings. In modelling whole countries, we have averaged over potentially important subnational differences. Our approach could be applied to subnational populations where the requisite data are available.

We also did not include effects due to COVID-19 and associated changes in mixing and access to care, and for this reason stopped model runs in 2019. COVID-19 typically caused decreases in TB notifications, but the true impact of the COVID-19 pandemic on TB epidemiology is as yet incompletely understood, with potential effects including reductions in community transmission, increases in household transmission, reduced access to care, increased mortality from COVID-19 affecting those at risk of incident TB or with prevalent TB^25–27^. As understanding of these features improves, these features could in future be included in our framework.

In the countries we considered, the high proportion of TB/HIV on ART emphasizes the importance of more intensive approaches to screening and prevention in these groups. PLHIV taking ART have accessed and remain in regular contact with health services; however, systematic screening for TB and TB preventive therapy should be strengthened. The high estimated ARTI and its pattern by age has implications for the measurement of TB transmission: measures have traditionally been constructed based on surveys in children, but may underestimate (re)infection risks in older age groups, as has previously been suggested^28,29^, and observed^30^.

Despite this high ARTI however, we find the proportion of TB incidence from infection within the last two years is below half overall in most settings, although higher in younger age groups. Better understanding this metric is important as it quantifies the proportion of incidence that could be averted by interventions to reduce transmission, such as active case finding and improved infection prevention and control^13^. Clustering metrics from high-resolution molecular epidemiology provide one approach to measurement, although there are few recently published studies from these countries. For Karonga, Malawi, 38% of strains have been reported as genetically linked to other samples within 5 years^31^. While TB incidence in children and adolescents could therefore respond rapidly to interventions that successfully reduce transmission, such interventions need to be supplemented by strategies that reduce progression from infection to disease (including vaccination), or mitigate the consequences of disease. Our finding that around half of transmission occurs from those aged 25-44 years highlights the importance of effectively engaging working-age adults with health services, including through work-based programmes. Age-specific contributions to transmission were highly consistent across the countries we considered due to similar patterns of mixing and relative TB prevalence. However, our sensitivity analysis considering children aged 10-14 years to be half as infectious as adults highlighted the potential for meaningful transmission from this group, which warrants more empirical investigation.

We have demonstrated the potential for calibrated transmission models to be used as a tool for TB burden estimation, and shown it is feasible to calibrate country-level TB models including age and HIV to empirical data while more fully accounting for uncertainty. In doing so, we have developed a number of technical innovations that should be of wider use, especially among those working on TB epidemics in high HIV prevalence settings. We provided a rigorous calculation of the proportion of TB incidence due to recent infection, finding substantial contributions from older infections are compatible with high annual TB infection risks in adults. Interventions that reduce TB transmission have the potential to produce rapid and substantial reductions in incidence in younger age groups, but will need to be combined with improved interventions addressing progression to have a major impact on the burden of TB in these settings.

## Methods

### Dynamics of population immunocompetence

We developed a simplified version of the deterministic compartmental AIM model of age- and sex-specific HIV incidence that is used in official UNAIDS estimates^14,15^. This model includes both sexes (*s*), 81 single-year age groups (*g*), 8 HIV and CD4 cell count categories (HIV-negative, and > = 500, 350–499, 250–349, 200–249, 100–199, 50–99, and < 50 cells per mm^3^, which we denote *h*),and 4 ART categories among PLHIV (no ART, 0-6 months, 7-12 months, 12+ months, which we denote *r* = 0,..,3) for a total of 5184 compartments. We assumed that a TB incidence rate ratio (IRR) at time *t* in each country applied to HIV-related states given by a two-parameter model (ρ controlling the interaction between CD4 decrement and TB risk; *α* controlling the protection from TB due to ART), *irr(t,s,g,h,r* | *α*,ρ*) = <α*^*r/3*^exp[ρΔ_h_]*>*, where < …> denotes an average over CD4 cell decrement below 1000 cells/mm^3^ (Δ_h_) in state *h*; *α* captures the protection from ART in states *r* = 0,..,3. We used a heuristic pursuit algorithm to achieve fit of this model to the HIV prevalence and ART coverage data used as inputs in the UNAIDS estimates. To facilitate inference, we used the AIM model to calculate mean IRRs and mortality for a model with simpler age (5-year age categories, *a*) and HIV/ART structure (*X* = *U* for HIV-negative, *H* for HIV-infected/ART-naive, *A* for on ART), for example defining *IRR*^*X*^*(t,s,a* | *α*,ρ*) = <irr(t,s,g,h,r* | *α*,ρ*)>*, where < …> denotes an average over the fine-grained age and HIV/ART states {*g,h,r*} corresponding to the state {*a, X*}. Because parameters *α* and ρ are uncertain and need to vary for inference, we emulated the dependence of these AIM-derived IRRs on *α* and ρ by running the model for a grid of *α* and ρ values and constructing a fast and accurate log-bilinear interpolation approximation to *IRR*^*X*^*(t,s,a* | *α*,ρ*)* for each country (see Supplementary Information).

### TB transmission model

We used the mean IRRs and HIV-associated mortality computed from this model, together with inputs derived from World Population Prospects demographic estimates^32^ to develop a TB transmission model with the simplified HIV/ART/demographic structure, that reproduced HIV and demographic trends. We used an established TB model structure with 6 states to represent: uninfected, fast-progressing latent infection, slow-progressing latent infection, TB disease, anti-TB treatment, and recovered from previous anti-TB treatment, which together with 17 age categories, both sexes and 3 HIV/ART stages gave 612 ordinary differential equations (see Supplementary Information). Our age-, sex-, and time-dependent TB IRRs were applied to all three pathways for incident TB (recent progression, non-recent reactivation, relapse after successful treatment). We used country-specific estimates of age-dependent mixing rates from Prem et al.^33^ in determining the force-of-infection, and assumed that children under 15 years were not infectious. We developed priors for the parameters of this model and its initial condition in 1970, based on established literature, including different progression rates and durations for children under 5, and odds ratios for detection in the 0–4 year and 5–14 year age categories (see Supplementary Information). As notification data are no longer reported to WHO stratified by sputum smear status, we did not represent it in the model and used case fatality ratios representing an average over smear status. The base detection model used a logit-normal prior with mean 0 and standard deviation 0.3 scaled onto the interval [0,0.9) to represent an initial case detection ratio (CDR) for each country, and a linear time trend in logit-space. We assumed no overdiagnosis, or undernotification of diagnosed TB.

### Data and likelihood

To capture the relative influence of TB notification and TB prevalence data, we used a hierarchical model that allowed the noise level for notifications (σ) to be learned from data. To use a single prior for σ across countries, we scaled by the maximum over yearly notifications. We used Gaussian approximations for the prevalence and notification likelihoods. The standard deviation for the prevalence likelihood (σ_*P*_) was based on the empirical precision of the estimate of bacteriologically-positive TB prevalence in those aged 15+ years (*P*_*e*_), so that, dropping constants, the data log-likelihood (*LL*) was given by:

1 $${LL}=-\frac{{\left({P}_{m}-{P}_{e}\right)}^{2}}{2{{\sigma }_{P}}^{2}}-\frac{{SSE}}{2{\sigma }^{2}}-n\,{{\log }}\left(\sigma \right)$$

where *P*_*m*_ is the corresponding transmission model estimate of TB prevalence, *n* is the number of years with notification data, and *SSE* is the sum-of-squares difference between TB notifications and corresponding model estimates, divided by the square of the maximum yearly notifications in each country. We chose an uncertain inverse-gamma prior for σ, which enabled us to integrate over σ in closed form, resulting in one fewer parameters to sample from and a marginal likelihood (*ML*):

2 $${ML}=-\frac{{\left({P}_{m}-{P}_{e}\right)}^{2}}{2{{\sigma }_{P}}^{2}}-\left[\alpha+\frac{n+1}{2}\right]{{\log }}\left({SSE}+b\right)$$

where *a* and *b* are the inverse-gamma hyperparameters (see Supplementary Information). In years where some of the notifications are age-stratified, we split estimates into stratified and unstratified components with a noise term consistent with the total, which results in a modified *SSE* term. While age-specific prevalence estimates are very uncertain, we incorporate this information in the data likelihood using relative rates of prevalence (15–24 years as the reference category) and a Gaussian approximation. Similarly, we calculated a fraction of routine notifications that were among PLHIV and its standard deviation using a random-effects meta-analysis, and used this as a target for the relevant years’ model average corresponding fraction.

We considered the 12 WHO high TB/HIV countries in Africa with a nationally representative TB prevalence survey. Of these, only South Africa had vital registration data. Death coding issues related to stigma mean that deaths recorded as due to TB in South Africa are a substantial overestimate of true TB deaths. We therefore did not include a mortality likelihood component.

### Bayesian inference

After integrating out σ, we were left with 20 parameters whose log prior densities were added to the likelihood. Each country was fitted separately using an ensemble slice sampling MCMC implementation^22^, using 50 chains and 2000 iterations. As sensitivity analyses, we recalibrated the model assuming: 10% of incident TB extrapulmonary; 30% of incident TB extrapulmonary; children aged 10–14 years 50% as infectious as adults.

### Proportion of TB incidence from recent transmission

The proportion of incidence due to recent transmission cannot be reliably calculated as the fraction of incidence from ‘fast’ progressing model compartments, especially when TB/HIV IRRs mean non-negligible fractions of ‘slow’ progressors will develop disease within 2 years. We carefully captured the proportion of incidence due to recent transmission within 2 years in 2019 by calculating incidence in an auxiliary copy of the differential equations with zero initial conditions, populated only by the (re)infection flows in the main model during 2017-2019 (see Supplementary Information).

### Reporting summary

Further information on research design is available in the Nature Portfolio Reporting Summary linked to this article.

## Supplementary information

Supplementary Information

Reporting Summary

## Data Availability

The collated data to reproduce these analyses are available at https://github.com/petedodd/estevez^34^.

## Code availability

The code to reproduce these analyses is available at https://github.com/petedodd/estevez^34^.
