# Supplementary material for: Transmission modeling to infer tuberculosis incidence prevalence and mortality in settings with generalized HIV epidemics
Source: Nat Commun. 2023 Mar 24;14:1639. doi: 10.1038/s41467-023-37314-1 (PMC10037365; doi:10.1038/s41467-023-37314-1)
Supplement: Supplementary file 1 — Supplementary Information [file 41467_2023_37314_MOESM1_ESM.pdf]

Transmission modeling to infer tuberculosis incidence prevalence and mortality in settings with generalized HIV epidemics

Peter J. Dodd[1], Debebe Adewo Shaweno[2], Chu-Chang Ku[2], Philippe Glaziou[3], Carel Pretorius[4], Richard J. Hayes[5], Peter MacPherson[6,7], Ted Cohen[8], and Helen Ayles[7,9]

[1]School of Health and Related Research, University of Sheffield, Sheffield, UK
[2]School of Public Health, Infectious Disease Epidemiology, Imperial College London, London, UK
[3]Global TB Programme, World Health Organization, Geneva, Switzerland
[4]Avenir Health, Glastonbury, CT, USA
[5]Department of Infectious Disease Epidemiology, Faculty of Epidemiology and Population Health, London School of Hygiene and Tropical Medicine, London, UK
[6]School of Health & Wellbeing, University of Glasgow, UK
[7]Department of Clinical Research, Faculty of Infectious and Tropical Diseases, London School of Hygiene and Tropical Medicine, London, UK
[8]Department of Epidemiology of Microbial Diseases, Yale School of Public Health, New Haven, CT, USA
[9]ZAMBART Project, Ridgeway Campus, University of Zambia, Lusaka, Zambia

Contents

1 Supplementary Methods **3**
1.1 Overview . . . 3
1.2 Model of demography and HIV/ART . . . 3
1.2.1 AIM model . . . 3
1.2.2 Quantities from AIM model & approximations . . . 5
1.3 TB model & priors . . . 10
1.3.1 Model structure . . . 10
1.3.2 Model equations . . . 11
1.3.3 Mixing and force-of-infection . . . 11
1.3.4 Initial state . . . 12
1.3.5 HIV/ART incidence & mortality . . . 12
1.3.6 Progression . . . 13
1.3.7 Durations, CFRs, self-cure . . . 13
1.3.8 Prevalence, detection, mortality . . . 13
1.3.9 Proportion recent . . . 14
1.3.10 Priors for parameters . . . 16
1.3.11 IRR heatmap . . . 17
1.4 Inference . . . 18
1.4.1 TB data & likelihood . . . 18
1.4.2 Prevalence likelihood . . . 18
1.4.3 Notifications: hierarchical likelihood & marginalization . . . 18
1.5 Sensitivity analyses . . . 21

2 Supplementary Results **23**
2.1 AIM HIV fits . . . 23
2.2 Inference outputs . . . 28
2.3 Corner plots . . . 29
2.4 Epidemiological plots with uncertainty . . . 41
2.5 Sensitivity analysis results . . . 45

1 Supplementary Methods

1.1 Overview

Figure 1 in the main article shows an overview of the modelling stages:

- Step 1: The simplified AIM model to describe HIV/ART, see Section 1.2.1
- Step 2-3: use of this model to generate HIV/ART-related inputs for a simpler model, Section 1.2.2
- Step 4: description of the TB transmission model, Section 1.3
- Step 5: description of data, likelihood, and inference, Section 1.4

1.2 Model of demography and HIV/ART

1.2.1 AIM model

The AIM model was developed by Avenir Health as part of the SPECTRUM package of tools for HIV burden estimation and programme planning, which are used in collaboration with countries to generate official UNAIDS HIV estimates.[1] The role of AIM specifically is to take take country-level estimates of HIV incidence and ART coverage and disaggregate these into more detailed demographic and HIV/ART states, in order to make projections of resource need and intervention impact. The model is run at a country level, and most of the data and parameters are country-specific.

The AIM model state space encompasses (characteristic, with number of categories in parentheses):

- **sexes (2)**: F, M
  (female/male)
- **ages (81)**: [0,1), [1,2),...,[79,80), [80,Inf)
  (single year age groups)
- **HIV (8)**: hiv-ve, >=500, 350-499, 250-349, 200-249, 100-199, 50-99, <50
  (CD4+ cell count per mm^3)
- **ART (4)**: none, [0,6)m, [7,12)m, [12,Inf)m
  (time on ART in months)

for a total dimension of $2 \times 81 \times 8 \times 4 = 5184$ (although some elements remain zero, eg those representing ART use among the HIV-negative population). The model is defined by specifying the dynamics for population count at time t, $N_t(s,a,h,r)$ in the compartment with sex s, age a, HIV state h, and ART state r. In the below, we

refer to the states in order for each characteristic above by integers (starting at 0). The dynamics satisfy a discrete version of the partial differential equation

$$\left(\frac{\partial}{\partial t}+\frac{\partial}{\partial a}\right)N_t(s,a,h,r)=\overbrace{b_t(s)\delta(a)}^{\text{births}}-\overbrace{\mu_t^0(s,a)N_t(s,a,h,r)}^{\text{background deaths}}+\overbrace{m_t(s,a)}^{\text{net migration}} \tag{1a}$$

$$-\chi_{h>0}\chi_{r=0}\times MR_t^H(s,a,h)\times\mu^H(s,a,h)N_t(s,a,h,r) \tag{1b}$$

$$-\chi_{h>0}\chi_{r>0}\times MR_t^A(r)\times\mu^A(s,a,h,r)N_t(s,a,h,r) \tag{1c}$$

$$-H(t)\pi_t^H(s,a,h)\left[\chi_{h=r=0}-\chi_{h>0}\right] \tag{1d}$$

$$-A(t)\pi_t^A(s,a,h)\left[\chi_{r=0}-\chi_{r=1}\right] \tag{1e}$$

$$-\chi_{r=0}\left[\chi_{h>0}R^H(s,a,h)N_t(s,a,h,r)-\chi_{h>1}R^H(s,a,h-1)N_t(s,a,h-1,r)\right] \tag{1f}$$

$$-\chi_{r>0}\left[R^A(r)N_t(s,a,h,r)-R^A(r-1)N_t(s,a,h,r-1)\right] \tag{1g}$$

where χ_C represents an indicator function for the condition C and $\delta(a)$ denotes the Dirac delta function. More detailed explanation follows below for each element of the right hand side.

Background demography (Eqn 1a): births, migrations and HIV-negative mortality were based on single-year data from the United Nations Population Division (World Population Prospects 2019 revision; WPP19), with life-tables adjusted to represent only non-HIV-associated mortality.

HIV mortality (Eqn 1b): applies only to those with HIV infection not on ART. The excess mortality rate $\mu^A(s,a,h)$ depends on sex, age, and CD4 count, but not calendar time. The mortality ratio $MR_t^H(s,a,h)$ specifies a country-specific time dependence and $[1-MR_t^H(s,a,h)]$ is taken to be the coverage of ART among all PLHIV at each sex/age/CD4 category.

ART mortality (Eqn 1c): applies only to those with HIV infection on ART. The excess mortality rate $\mu^A(s,a,h)$ depends on sex, age, CD4 count at ART initiation, and time-on-ART, but not calendar time. The mortality ratio $MR_t^A(r)$ specifies a country-specific time dependence.

HIV incidence (Eqn 1d): the number of HIV infections at each timestep is calculated with a heuristic pursuit approach developed for this application. $H(t)$ is adjusted at each time step in the Euler method solver for the dynamics so that the prevalence of HIV in the total population matches data. In particular, the number of new HIV infections H_t^{model} at timestep t (approximating $H(t)$ in the Euler scheme) is taken as

$$H_t^{model}=\mathscr{F}\times\max(H_{t-1}^{data}-H_{t-1}^{model},0)$$

where H_{t-1}^{data}, H_{t-1}^{model} are the number of prevalent HIV infections at the previous timestep in the target data and model, respectively, and $\mathscr{F}<1$ is a tuning factor adjusted by hand to achieve good fits while remaining stable. Target data were linearly interpolated between years. See `AIMdynInc.R` source code in the repository for details.

The number of HIV infections are distributed according to

$$\pi_t^H(s,a,h) \propto \sigma(s)\gamma(s,a)\kappa(a,h)N_t(s,a,0,0) \tag{2}$$

with a sex-specific risk ratio ($\sigma(s)$), a sex-specific set of relative risks by age ($\gamma(a,s)$), and new infections flow into an age-dependent distribution of CD4 count categories ($\kappa(a,h)$). HIV incidence only occurs in those aged ≥ 15 years; we neglect paediatric HIV.

ART initiation (Eqn 1e): the number of ART initiations at each timestep is calculated with a heuristic pursuit approach (as for HIV; see above), adjusting $A(t)$ so that the prevalence of ART among people living with HIV matches data on ART coverage among PLHIV, pre-smoothed as a 3 year moving average. The number of ART initiations are distributed among those not on ART with CD4 count below 500 cells/mm^3 proportional to the mean of HIV mortality and population count, ie

$$\pi_t^A(s,a,h) \propto \chi_{h>0}\left(\omega\mu^H(s,a,h)+1-\omega\right)N_t(s,a,h,0)/2 \tag{3}$$

where $\omega = 0.2$.

HIV progression (Eqn 1f): applies only to those with HIV infection not on ART and is not dependent on calendar time. The rate at which CD4 categories advance into the next category ($R^H(s,a,h)$) depends on sex, age and the current CD4 category.

ART progression (Eqn 1g): applies only to those with HIV infection on ART and is not dependent on calendar time. The rates $R^A(r)$ are taken as the inverse of the incremental time on ART associated with state r. We take $R^A(3) = 0$. Note that the state h does not advance on ART; for those on ART the value of this state represents the CD4 category at which ART was initiated. We simplify the full AIM model here by neglecting ART loss to follow-up.

The simplified AIM model was implemented in the R programming language.[2] The model is initialized using WPP19 demographic estimates and zero HIV prevalence. It is run with a 1/10th of a year time step. Code for this model is part of the GitHub repository for this project (`https://github.com/petedodd/estevez`) and the AIM data required to run the model was extracted from the UNAIDS country fits available via AIM SPECTRUM web (`https://aim.spectrumweb.org/`). The data to run the model for these countries is included in the repository. Example outputs from this model are shown in Section 2.1.

1.2.2 Quantities from AIM model & approximations

Quantities calculated

Our simplified model uses 5 year age groups ([0,5), [5,10),...,[75,80), [80,Inf)). We use output from the AIM model for each country in order to calculate the following quantities that serve as inputs for our simplified model:

- $\bar{\mu}_t^H(s,a)$ - mean mortality in HIV+/ART-
- $\bar{\mu}_t^A(s,a)$ - mean mortality in HIV+/ART+

- $IRR_t^H(s,a)$ - mean incidence rate ratio for TB in HIV+/ART-
- $IRR_t^A(s,a)$ - mean incidence rate ratio for TB in HIV+/ART+

The average mortalities for each 5-year age group are calculated as

$$\bar{\mu}_t^H(s,a) = \frac{\sum_{a'\in a,h>0}\{\mu^0(s,a)+\mu^H(s,a,h)\}N_t(s,a,h,0)}{\sum_{a'\in a,h>0} N_t(s,a,h,0)}, \tag{4}$$

and

$$\bar{\mu}_t^A(s,a) = \frac{\sum_{a'\in a,h>0,r>0}\{\mu^0(s,a)+\mu^A(s,a,h,r)\}\mu^A(s,a,h,r)N_t(s,a,h,r)}{\sum_{a'\in a,h>0,r>0} N_t(s,a,h,r)}. \tag{5}$$

The explicit time evolution captures the dynamics of the underlying CD4 cell count distribution and ART durations in each sex and age-group. Figure 1 shows an example snapshot of population CD4 distribution.

Similarly, the mean incidence rate ratios for TB capture the evolution of the average immuno-competence in each sex and age-group. These are calculated as

$$IRR_t^H(s,a;\rho) = \frac{\sum_{a'\in a,h>0} irr(h;\rho)N_t(s,a,h,0)}{\sum_{a'\in a,h>0} N_t(s,a,h,0)}, \tag{6}$$

and

$$IRR_t^A(s,a;\rho,\alpha) = \frac{\sum_{a'\in a,h>0,r>0} \alpha^{r/3} irr(h;\rho)N_t(s,a,h,r)}{\sum_{a'\in a,h>0,r>0} N_t(s,a,h,r)}. \tag{7}$$

Here, the quantity $irr(\rho)$ is the mean incidence rate ratio (IRR) in CD4 category h modelled as

$$irr(h;\rho) = \frac{1}{[CD4_+(h) - CD4_-(h)]}\int_{CD4_-(h)}^{CD4_+(h)} \mathrm{d}\Delta\ \exp\left[\rho(1000-\Delta)\right], \tag{8}$$

ie the mean over CD4 count decrements Δ between the upper and lower points defining category h ($CD4_+(h)$ and $CD4_-(h)$, respectively) of and exponentially increasing IRR (ie $e^{\rho\Delta}$). This exponential model of IRR is based on previous work.[3, 4] In Eqn 7, this is multiplied by $\alpha^{r/3}$ to model the protection from TB due to ART. For established ART ($r = 3$), the hazard ratio α is based on systematic review, and the use of $\alpha^{1/3}$ during the first 6 months on ART and $\alpha^{2/3}$ during months 6-12 on ART is designed to qualitatively capture the improvement in protection after starting ART.[5, 6]

Approximations

Because parameter ρ and α are treated as random variables with uncertainty in our inference approach, we need a way to approximate the dependence in $IRR_t^H(s,a;\rho)$ and $IRR_t^A(s,a;\rho,\alpha)$ on ρ and α to avoid re-running the full AIM model at each likelihood evaluation in an inference algorithm. For each country, we evaluated the IRR

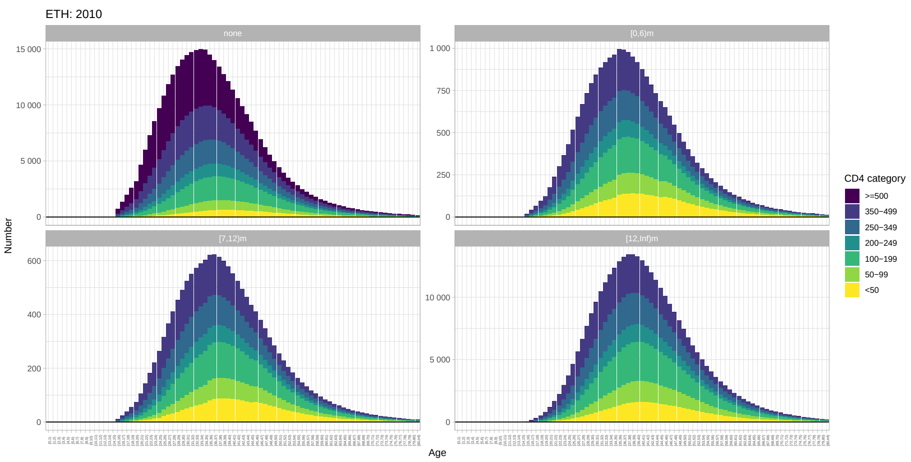

Figure 1: Example of population CD4 and ART distributions (Ethiopia in 2010)

time series for a grid of (ρ,α) parameter values, namely at $(\rho,\alpha)\in G=\{0.1,0.15,\ldots,0.8\}\times\{0.1,0.2,\ldots,0.8\}$. We then used bilinear interpolation for the logarithm of the IRRs:

$$\log(IRR_t^X(s,a;\rho)))\approx\frac{1}{A}\begin{bmatrix}\rho_2-\rho\\ \rho-\rho_1\end{bmatrix}^T\begin{bmatrix}\ell_t^X(s,\rho_1,\alpha_1) & \ell_t^X(s,\rho_1,\alpha_2)\\ \ell_t^X(s,\rho_2,\alpha_1) & \ell_t^X(s,\rho_2,\alpha_2)\end{bmatrix}\begin{bmatrix}\alpha_2-\alpha\\ \alpha-\alpha_1\end{bmatrix}, \quad (9)$$

where the sex $s\in\{M,F\}$, ART status $X\in\{H,A\}$, $\ell_t^X(s,\rho,\alpha)=\log(IRR_t^X(s,a;\rho)))$, A is the grid area (ie $(\alpha_2-\alpha_1)\times(\rho_2-\rho_1)$), and (ρ_i,α_j) are the corners in G defining the smallest rectangle containing (α,ρ).

The performance of this approximation is shown in Figure 2. Figure 2a shows the approximation vs exact calcualtion for a single parameter set, by time and age group. Figure 2b shows an exploration across different parameter values. A latin hypercube sample of 50 parameter values was sampled across the parameter ranges for ρ and α. Exact and approximate IRRs were computer across ages, sexes and ART status. Only time periods with non-zero HIV or ART were retained. Over this sample of 77,000 comparisons, the mean absolute error for IRR was a maximum for Malawi at 1.1% and below 1% in all other countries.

Simplified model demography & HIV/ART

In order to simplify the description of the approach to demography & HIV/ART we present this without the TB model structure. The age, sex, and HIV/CD4 & ART structure for AIM described above in Section 1.2.1 is reduced to a structure with both sexes, 5-year age groups, and 3 HIV/ART strata:

- **sexes (2)**: F, M
  (female/male)

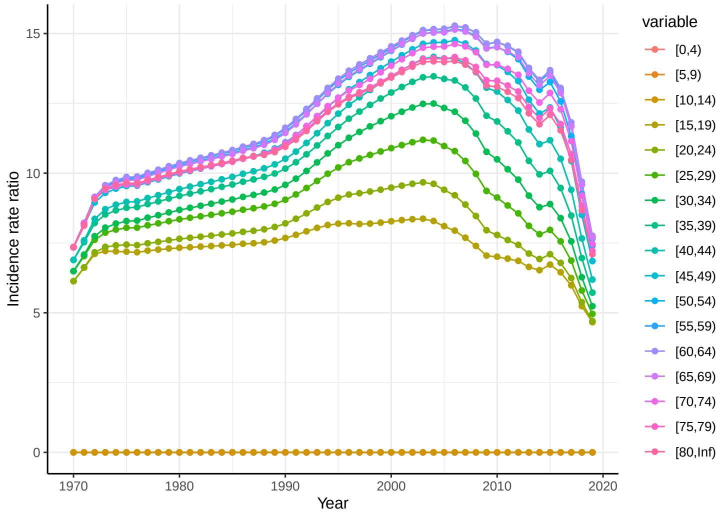

(a) IRR for MWI/F/ART-ve, $(\rho, \alpha) = (0.36, 0.33)$. (Lines - exact; dots - approximation)

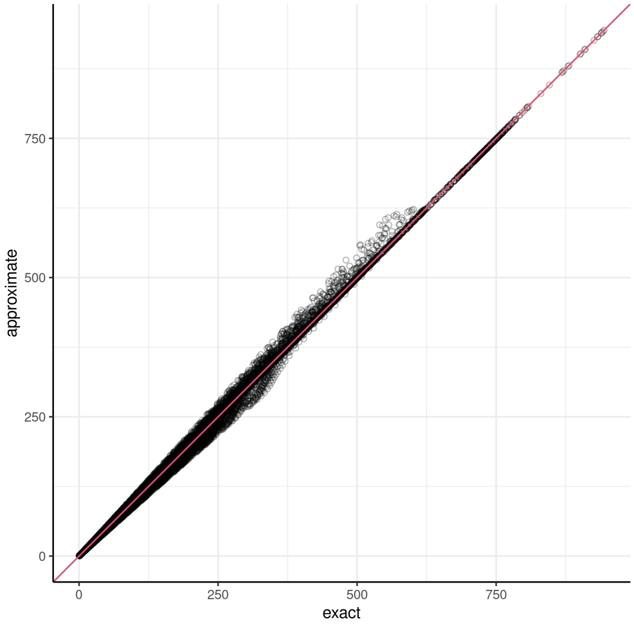

(b) Exact vs approximation for a latin hypercube sample of 50 (α, ρ) pairs, across times, ages, sexes, and ART status

Figure 2: Performance of bilinear interpolation for incidence rate ratio

- **ages (17)**: [0,5), [5,10),...,[75,80), [80,Inf)
  (5 year age groups)
- **HIV (2)**: hiv-ve, hiv+ve
  (HIV uninfected/HIV infected)
- **ART (2)**: art-ve, art+ve
  (no ART/on ART)

for a total dimension of $2 \times 17 \times 3 = 102$ (although the element representing ART use among the HIV-negative population remain zero). This is approximately a factor of 500× smaller than the full AIM model. As above, we will sometimes refer to each state by index in order (starting at 0). At time t, age a, and for sex s, define $X_t(s,a)$ to be the number of people without HIV infection, $H_t(s,a)$ the number of PLHIV not on ART, and $A_t(s,a)$ the number of PLHIV on ART.

$$\frac{\mathrm{d}}{\mathrm{d}t} X_t(s,a) = b_t(s) - \bar{\mu}_t^0(s,a) X_t(s,a) \tag{10a}$$

$$- h_t(s,a) X_t(s,a) \tag{10b}$$

$$+ \chi_{a>0} r X_t(s,a-1) - r X_t(s,a) \tag{10c}$$

$$\frac{\mathrm{d}}{\mathrm{d}t} H_t(s,a) = -\bar{\mu}_t^H(s,a) H_t(s,a) - \bar{\mu}_t^0(s,a) H_t(s,a) \tag{10d}$$

$$+ h_t(s,a) X_t(s,a) - c_t(s,a) H_t(s,a) \tag{10e}$$

$$+ \chi_{a>0} r H_t(s,a-1) - r H_t(s,a) \tag{10f}$$

$$\frac{\mathrm{d}}{\mathrm{d}t} A_t(s,a) = -\bar{\mu}_t^0(s,a) A_t(s,a) - \bar{\mu}_t^A(s,a) A_t(s,a) \tag{10g}$$

$$+ c_t(s,a) H_t(s,a) \tag{10h}$$

$$+ \chi_{a>0} r A_t(s,a-1) - r A_t(s,a) \tag{10i}$$

Ageing (Eqns 10c, 10f, 10i) Ageing from one 5-year age group to the next is modelled (conventionally) as occurring with a rate $r = 1/5$ per year.

HIV/ART incidence (Eqns 10b, 10e, 10h) The number of HIV infections and ART initiations is derived by aggregating that calculated by the pursuit heuristics in our AIM model to fit HIV prevalence and ART coverage data.

Mortality/births (Eqns 10a, 10d, 10g)

Mortality rates were calculated based on WPP19 timeseries of population sizes and births.

For ODE population dynamics, with birth rate $B(t)$, age categories $a = 0, \ldots,$ and ageing rate r

$$\partial_t N(t,a) = B(t)\delta_a + r.N(t,a-1) - \omega_t(a).N(t,a),$$

we can rewrite to define

$$\omega_t(a) = \quad r.\{N(t,a-1)/N(t,a)\} - \partial_t \log(N(t,a))$$

$$\omega_t(a) \stackrel{\text{def}}{=} \quad r.\rho(t,a-1) - \delta(t,a), \tag{11}$$

i.e. defining $\delta(t,a) = \partial_t \log(N(t,a))$ & $\rho(t,a) = N(t,a)/N(t,a+1)$ for $a > 0$ and $\rho(t,0) = B(t)/N(t,0)$. We calculate the derivative $\delta(t,a)$ from a smoothing spline fit to the log-population, for men and women separately. With this notation,ω includes both ageing and mortality as $\omega_t(a) = \mu_t(a) + r$.

To adapt these results to Eqns 10a-10i, we recalculate the background mortality $\bar{\mu}^0_t$ accounting for HIV/ART mortality derived for example from Eqns 4&5, to give the correct total mortality from Eqn 11

$$\bar{\mu}^0(t,s,a) = (\mu(t,s,a)N(t,s,a) - \bar{\mu}^H(t,s,a)H(t,s,a) - \bar{\mu}^A(t,s,a)A(t,s,a))/N(t,s,a).$$

1.3 TB model & priors

1.3.1 Model structure

The model includes 6 TB strata:

- S - uninfected by *M. tuberculosis*,
- E - infected, with 'fast' progression rate applied,
- L - infected, with 'slow' progression rate applied,
- I - prevalent & infectious TB disease,
- T - patients receiving anti-TB treatment (assumed uninfectious),
- R - previously treated patients at additional risk of relapsing to TB disease.

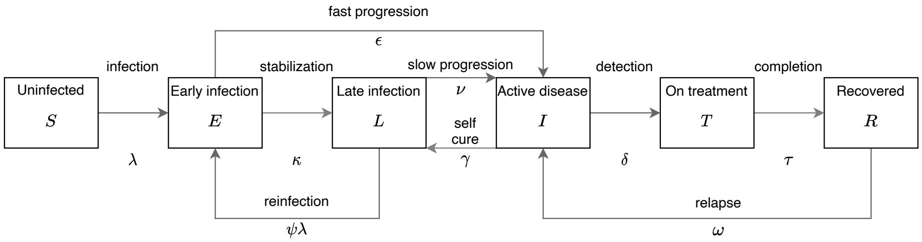

Figure 3: TB transmission model structure. Not shown: HIV/ART structure; age/sex structure; reinfection from Recovered; birth and background mortality.

1.3.2 Model equations

Using a superscript $i \in \{X, H, A\}$ to index the HIV/ART stratum of each state, the ODEs for the TB transmission model are

$$\frac{\mathrm{d}S^i}{\mathrm{d}t} = b^i + (1-\theta)\tau T^i - (\mu^i + \lambda)S^i - \sum_j h^{ij} S^j - r[S^i(s,a) - \chi_{a>0} S^i(s,a-1)] \tag{12a}$$

$$\frac{\mathrm{d}E^i}{\mathrm{d}t} = \lambda S^i + \psi^i \lambda L_i - (\mu^i + \kappa + \varepsilon^i)E^i - \sum_j h^{ij} E^j - r[E^i(s,a) - \chi_{a>0} E^i(s,a-1)] \tag{12b}$$

$$\frac{\mathrm{d}L^i}{\mathrm{d}t} = \kappa E^i + \gamma^i I_i - (\mu^i + v^i + \psi^i \lambda)L^i - \sum_j h^{ij} L^j - r[L^i(s,a) - \chi_{a>0} L^i(s,a-1)] \tag{12c}$$

$$\frac{\mathrm{d}I^i}{\mathrm{d}t} = \varepsilon^i E^i + v^i L^i + \omega^i R^i - (\mu^i + \mu^i_{TB} + \gamma^i + \delta^i)I^i - \sum_j h^{ij} I^j - r[I^i(s,a) - \chi_{a>0} I^i(s,a-1)] \tag{12d}$$

$$\frac{\mathrm{d}T^i}{\mathrm{d}t} = \delta^i I^i - (\tau + \mu^i)T^i - \sum_j h^{ij} T^j - r[T^i(s,a) - \chi_{a>0} T^i(s,a-1)] \tag{12e}$$

$$\frac{\mathrm{d}R^i}{\mathrm{d}t} = \theta\tau T^i - (\omega^i + \mu^i)R^i - \sum_j h^{ij} R^j - r[R^i(s,a) - \chi_{a>0} R^i(s,a-1)]. \tag{12f}$$

Here, we have suppressed the time- and sex/age-dependence of $S^i_t(s,a)$ etc for clarity, except in the ageing terms [shown in square brackets].

The 6 TB states, 2 sexes, 17 ages and 3 HIV/ART strata make for a total of $6 \times 2 \times 17 \times 3 = 612$ states and corresponding dynamical equations. The model was implemented using the odin package in R,[7] with the dynamics being compiled via C for speed and solved with default routines provided by the deSolve package.[8]

1.3.3 Mixing and force-of-infection

Writing

$$Z_a(t) = \sum_s [S_t(s,a) + E_t(s,a) + L_t(s,a) + I_t(s,a) + T_t(s,a) + R_t(s,a)] \tag{13}$$

for the total population in each group, we define the force-of-infection in the above equations by

$$\lambda_a(t) = \beta \sum_{i,s,a'} M_{a,a' \in \mathbb{A}} \frac{I^i_t(s,a')}{Z_{a'}(t)}. \tag{14}$$

where $\mathbb{A}$ are the adult age categories that exclude $[0,5)$, $[5,10)$, and $[10,15)$ since we assume children <15 years are not infectious.

Mixing matrix for each country based on the results of the hierarchical modelling of social contact studies presented in Prem et al [9]. We used the total contacts, and rescaled so that the maximum contact rate for any age group was 1.

1.3.4 Initial state

We use a single parameter (Λ_0) to define a heuristic model initial state in 1970, designed to minimize transient dynamics but allow for different initial intensities of TB epidemic. In particular, we take

$$
\begin{aligned}
f_0 &= (\omega + \nu + \psi\Lambda_0)/\kappa \\
f_L(a) &= 1 - \exp(-a \times \Lambda_0) \\
d_0 &= 2\nu D^X(1-K).
\end{aligned}
$$

Then, with $Z(s,a)$ the total population in 1970 by sex s and age a, we take

$$
\begin{aligned}
S^X(s,a) &= Z(s,a)[1 - f_L(a)] \\
E^X(s,a) &= Z(s,a) f_L(a) f_0/2 \\
L^X(s,a) &= Z(s,a) f_L(a)[1 - f_0/2 - 4d_0/3] \\
I^X(s,a) &= Z(s,a) d_0 f_L(a) \\
T^X(s,a) &= Z(s,a) d_0 f_L(a)/3 \\
R^X(s,a) &= Z(s,a) f_L(a) f_0/2.
\end{aligned}
$$

All states corresponding to PLHIV were initially set to zero.

1.3.5 HIV/ART incidence & mortality

HIV/ART incidence The matrix of HIV/ART incidence is defined by

$$
h_t^{ij}(s,a) = \begin{array}{c} \\ X \\ H \\ A \end{array} \begin{array}{c} \begin{array}{ccc} X & H & A \end{array} \\ \begin{pmatrix} -h_t(s,a) & 0 & 0 \\ h_t(s,a) & -\alpha_t(s,a) & 0 \\ 0 & \alpha_t(s,a) & 0 \end{pmatrix} \end{array} \tag{15}
$$

where $h_t(s,a)$ and $\alpha_t(s,a)$ derived from the AIM model.

Mortality The time/sex/age-dependent mortality rates for PLHIV (μ^H & μ^A) are determined from the AIM model; the background mortality in HIV-negative individuals is determined to match total mortality from Eqn 11 once HIV/ART and TB mortality are accounted for.

1.3.6 Progression

The two-compartment model of progression from infection to disease and associated parameters are based on that preferred by Ragonnet et al.[10], except for children under 5 years of age, where fast progression is based on Martinez et al.[11]

Influence of HIV The hazards determining the three routes to TB incidence (ε, υ, and ω) are each multipltied by the corresponding incidence rate ratios from Eqns 6&7

$$\varepsilon^i(t,s,a) = IRR^i_t(s,a) \times \varepsilon$$
$$\upsilon^i(t,s,a) = IRR^i_t(s,a) \times \upsilon$$
$$\omega^i(t,s,a) = IRR^i_t(s,a) \times \omega,$$

for $i \in \{H, A\}$.

1.3.7 Durations, CFRs, self-cure

The timescale for untreated TB is set by duration parameters (D^i_a) based on a review of the pre-chemotherapy literature,[12] and modifications for PLHIV (see below). For children under 15 years of age, an arbitrary duration of 6 months is used. Prevalence in this age group is essentially irrelevant since they are assumed not to be infectious and there is no prevalence data. Correspondingly, there is no child-specific evidence on the duration of untreated TB, but TB in young children is felt to be more rapid in clinical progression than in adults.

Durations and case-fatality ratios (Φ^i) without treatment are used to define self-cure rates via

$$\gamma^i = \frac{1-\Phi^i}{D^i_a}, \tag{16}$$

with TB-related hazards of mortality then being $\mu^i_{TB} = \Phi^i / D^i_a$.

1.3.8 Prevalence, detection, mortality

Prevalence The prevalence of bacteriologically-positive TB in adults (age 15+ years) using the model states I in the numerator, and relevant population sums (Z) in the denominator.

Extra-pulmonary TB To account for the fact that not all TB is pulmonary, we assume a fixed factor $\eta = 0.83$ of incidence and notifications (and associated mortality) is pulmonary, based on an analysis of global notification data.[13] Incidence, notifications, and mortality reported divide model outputs by this factor to inflate for extra-pulmonary TB, which is not considered explicitly (and therefore not considered infectious) in the TB transmission model.

Detection We define a dynamic probability of TB detection via a linear trend in logit space:

$$\text{logit}\left(\frac{p(t)}{0.9}\right) = \text{logit}(K) + c(t - t_0),$$

where $t_0 = 1970$ the start time for the model. This probability is capped at 0.9 both for realism, and because higher values can imply extreme rates resulting in numerical instability in the ODE solver.

This probability $p(t)$ is used to define the rates of detection for each age group as

$$\delta^i_a(t) = \text{OR}_a \times \frac{p(t)}{1 - p(t)} \times \frac{1}{D^i_a}$$

The odds ratios for detection are 1 for adult age categories (≥ 15), and take values OR_{04} and OR_{514} for a in the corresponding age ranges.

Notifications All this means that notifications in each group are outputted from the model as

$$\hat{N}^i_t(a) = \sum_s \delta^i_a(t) I^i_t(s,a)\eta^{-1}. \tag{17}$$

Mortality Mortality from TB, $m^i_t(s,a)$, is defined as the sum of deaths on treatment and those without treatment

$$m^i_t(s,a) = 2\theta T^i(s,a)\eta^{-1} + \Phi^i \frac{I^i_t(s,a)}{D^i_a}\eta^{-1},$$

the factor of 2 reflecting a 0.5 year treatment duration.

1.3.9 Proportion recent

TB incidence derivces from 3 routes: from the fast-progressing latent infection state (E); from the slow-progressing latent infection state (L); from the recovered state (R). The proportion of incidence due to (re)infection within a certain timeframe, T (usually taken as 2 years) is not just the proportion of incidence from the fast state, as all 3 routes will contribute recent incidence to varying degrees. This is further complicated by the influence of HIV, which implies that substantial contributions to recent incidence may be made by the slow-progressing latent infection state due to the high IRRs. We describe here how to calculate the proportion of incidence due to recent infection for an ODE model such as that used in this work.

Let τ be the time-since-(re)infection and let

$$Y_b(t,a,\tau)$$

be all the model states, with index b denoting sex and TB/HIV state. The dynamics are of the form

$$\left(\frac{\partial}{\partial t} + \frac{\partial}{\partial \tau}\right) Y_b(t,a,\tau) = \overbrace{r.Y_b(t,a-1,\tau)}^{\text{ageing}} + \overbrace{\sum_c M_{bc} Y_c(t,a,\tau)}^{Y-\text{transitions}} \tag{18}$$

with the boundary condition

$$Y_b(t,a,0) = y_b(t,a) \tag{19}$$

representing inflows due to (re)infection. The coefficients M_{bc} depend on t and a, but not τ, and we take them to be those from eqns 12a-12f except with zero force-of-infection. We consider the inflow boundary condition to be zero except during the interval $[t_r - T, t_r]$ when we set it to the (re)infection flows in the ODEs from the main model. We take the initial conditions to be zero ($Y_b(0,a,\tau) = 0$).

Define

$$Y_b^R(t,a) = \int_0^{\mathrm{T}} \mathrm{d}\tau \; Y_b(t,a,\tau) \tag{20}$$

to be the population in state Y_b that has been (re)infected within time T. To calculate $Y_b^R(t_r,a)$ at some reference time t_r, Then because of the τ-independence of the coefficients, and using eqns 18 & 20 the $Y_b^R(t,a)$ satisfy ODEs

$$\begin{aligned}
\tfrac{\mathrm{d}}{\mathrm{d}t} Y_b^R(t,a) &= \textstyle\int_0^{\mathrm{T}} \mathrm{d}\tau \, \frac{\partial Y_b(t,a,\tau)}{\partial t} \\
&= -\textstyle\int_0^{\mathrm{T}} \mathrm{d}\tau \, \frac{\partial Y_b(t,a,\tau)}{\partial \tau} + r.Y_b^R(t,a-1) + \sum_c M_{bc} Y_c^R(t,a) \\
\tfrac{\mathrm{d}}{\mathrm{d}t} Y_b^R(t,a) &= y_b(t,a) + r.Y_b^R(t,a-1) + \textstyle\sum_c M_{bc} Y_c^R(t,a)
\end{aligned} \tag{21}$$

where the last equality uses the boundary condition eqn 19 and the fact that $Y_b(t,a,\mathrm{T}) = 0$ for $t \in [t_r - \mathrm{T}, t_r]$ because τ and t move together (ie the characteristics have speed 1) and the inflow was zero until time $t = t_r - T$, implying no density has reached this point yet.

In summary, we can calculate the proportion of incidence that is recent by duplicating the main model ODEs (eqns 12a-12f) except that the (re)infection flows are those in the main model multiplied by an indicator function that is zero except for the T years before the reference time ($\chi(t \in [t_r - \mathrm{T}, t_r])$), and the initial conditions are zero. These 'cloned' equations (eqns 21) are thus only populated via (re)infection within period T, and so the incidence calculated from them is that due to transmission within this period.

1.3.10 Priors for parameters

Table 1: Priors and sources for model parameters.
Units: y^{-1} = per year, y = year.
Abbreviations: LTBI=latent TB infection; IRR=incidence rate ratio; CDR=case detection ratio; OR=odds ratio; CFR=case fatality ratio.
§: scaled to $(0, 0.9)$.
†: on a scale set by the maximum yearly notification in a country.
‡: the WHO age/sex disaggregation uses a paediatric-specific prior based on [14] and consistency with data.

Parameter	Meaning	Distribution	Source
Transmission			
β	Effective contact rate, y^{-1}	LogNormal(log(10),0.75)	assumption
ψ	Partial infection protection with LTBI	Beta(20.7,77.9)	Andrews[15]
Λ_0	Initial condition parameter, y^{-1}	LogNormal(log(3e-2),0.75)	assumption
Progression			
κ	Stabilization rate, y^{-1}	LogNormal(0.62, 0.068)	Ragonnet[10]
ε	Fast progression rate, y^{-1}	LogNormal(-2.837,0.32)	Ragonnet[10]
ε_{04}	Progression children <5 years, y^{-1}	Beta(5.153,21.967)	Martinez[11]
ν	Slow progression rate, y^{-1}	LogNormal(-6.89,0.58)	Ragonnet[10]
ω	Relapse rate, y^{-1}	LogNormal(-3.95,0.27)	Crampin[16]
HIV/ART IRRs			
ρ	CD4 IRR parameter	LogNormal(-1.02,0.219)	Ellis[3]
α	ART protection	LogNormal(-1.05,0.115)	Suthar[17]
Detection			
K	Case detection ratio (initial)	LogitNormal(0,0.3)	assumption§
c	Log rate of CDR change, y^{-1}	Gamma(k=0.5,θ=4e-2)	assumption
OR_{04}	OR for detecction children <5 years	LogNormal(-0.100,0.626)	WHO estimates†
OR_{514}	OR for detecction children 5-14 years	LogNormal(-0.567,0.458)	WHO estimates†
Transmission			
D^X	Duration of untreated TB, y	LogNormal(1.1,0.2)	log(3) & assumed SD [12]
D^H	Duration of untreated TB (HIV+), y	Gamma(7.374,0.065)	Ku[18]
Case fatality ratios			
θ	CFR on TB treatment	Beta(87.55,2.71)	synthesis in WHO appendix[13]
Φ^X	CFR for untreated TB (HIV-)	Beta(25.48,33.78)	synthesis in WHO appendix[13]
Φ^H	CFR for untreated TB (HIV+/ART-)	Beta(23.68,6.68)	synthesis in WHO appendix[13]
Φ^A	CFR for untreated TB (HIV+/ART+)	Beta(11.88,12.37)	synthesis in WHO appendix[13]
Notification noise			
σ	Notification noise term	InvGamma(α=5,β=0.1^2)	assumption‡

1.3.11 IRR heatmap

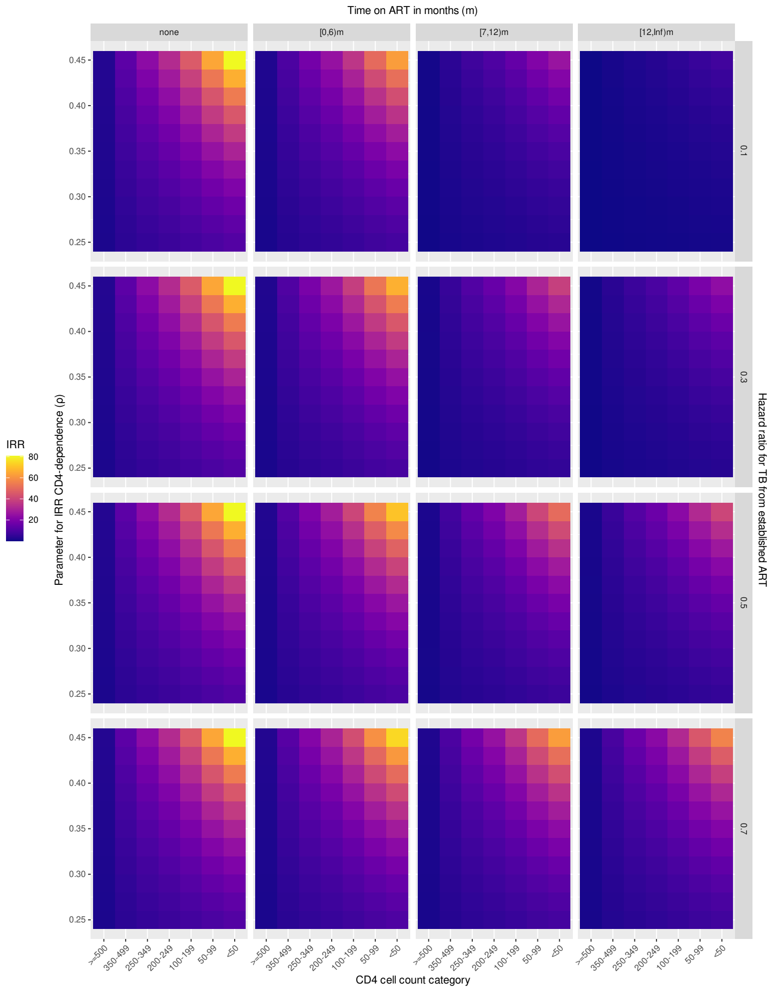

Figure 4: Heatmap showing the incidence rate ratio (IRR) for TB implied by the parameters α (rows) and ρ (y-axis) in each CD4 cell-count category (x-axis) and ART category (columns) of the AIM model.

1.4 Inference

1.4.1 TB data & likelihood

The total log-likelihood is the sum of the data log-likelihood and the log-prior:

$$LL = LL_{\text{data}} + LL_{\text{prior}}. \tag{22}$$

For the countries we consider, there is no mortality data that can be used in a straightforward way: only South Africa has vital registration data, and deaths coded to TB in this data are thought to overestimate TB deaths by a substantial amount. For this work, we therefore make use of notification and prevalence data, so that the data log-likelihood decomposes as:

$$LL_{\text{data}} = LL_{\text{notifications}} + LL_{\text{prevalence}}. \tag{23}$$

1.4.2 Prevalence likelihood

For the prevalence log-likelihood, we use

$$LL_{\text{prevalence}} = -\frac{(P_t - \hat{P}_t)^2}{2\sigma_P^2} - \sum_a \frac{(PR_a - \hat{PR}_a)^2}{2\sigma_a^2}. \tag{24}$$

In the first term, P_t is the prevalence of bacteriologically-positive TB in those aged ≥ 15 years from the prevalence survey, and $\hat{P}_t$ the corresponding model prediction in that year. The parameter σ_P is derived from the precision reported for the prevalence estimate (ie from the reported confidence interval, which takes into account the prevalence survey design).

The second term takes into account the age pattern in the prevalence survey if reported. To avoid double-counting information on the level of prevalence, we work with the prevalence ratios with respect to the reference age group of 15-24 years. We calculate prevalence ratios for age groups 25-34 years, 35-44 years, 45-54 years, 55-64 years, and 65+ years, and corresponding variances σ_a from the TB prevalence survey age-stratified point estimates and confidence intervals. $\hat{PR}_a$ is the model-predicted prevalence ratio in age group a.

1.4.3 Notifications: hierarchical likelihood & marginalization

We first break the notification log-likelihood into two parts

$$LL_{\text{notifications}} = LL_n + LL_h, \tag{25}$$

where LL_n is a log-likelihood capturing the level of notifications (and pattern by age), and LL_h is a log-likelihood capturing the pattern of notifications with respect to HIV.

Considering first total notifications at each time, N_t, the relevant log-likelihood is taken to be

$$LL_n = -\sum_t \frac{(N_t - \hat{N}_t)^2}{2\sigma^2} - \frac{n}{2}\log(\sigma), \tag{26}$$

where $\hat{N}_t$ is the model prediction of the number of notifications in a given year from eqn 17, n is the number of years with data, and σ is the noise level that captures a range of unobserved processes that cause notifications vary from year to year.

We model the noise with a prior so that the data inform this parameter. In fact, to facilitate inference across different countries, we assume that notifications have a noise which scales with peak notifications in a country, F, and therefore that the likelihood is

$$L_n = \prod_t \mathcal{N}(N_t - \hat{N}_t, \sigma F) = \frac{1}{(2\pi\sigma^2 F^2)^{n/2}} \exp\left[-\frac{E}{\sigma^2}\right] \tag{27}$$

where

$$E = \sum_t \frac{(N_t - \hat{N}_t)^2}{2F^2}$$

For σ we use an inverse-gamma prior:

$$P_\sigma(\sigma^2) = \frac{\beta^\alpha}{\Gamma(\alpha)} (1/\sigma^2)^{\alpha+1} \exp\left(-\beta/\sigma^2\right). \tag{28}$$

This enables us to marginalize the notification likelihood for σ:

$$\begin{aligned} M = \int_0^\infty \mathrm{d}\sigma\, P_\sigma(\sigma) L_n(\sigma) &= \frac{\beta^\alpha}{\Gamma(\alpha)} \frac{1}{(2\pi F^2)^{n/2}} \int_0^\infty \mathrm{d}\sigma\, \sigma^{-2(\alpha+1+n/2)} \exp\left[-\frac{(E+\beta)}{\sigma^2}\right] \\ &= \frac{\beta^\alpha \Gamma\left(\alpha + \frac{1+n}{2}\right)}{\Gamma(\alpha)(2\pi F^2)^{n/2} \times 2(E+\beta)^{\alpha+(1+n)/2}} \end{aligned}$$

using

$$\int_0^\infty \mathrm{d}x\, x^{-A} e^{-B/x^2} = \frac{1}{2B^{(A-1)/2}} \Gamma\left(\frac{A-1}{2}\right)$$

where the Gamma function is defined by

$$\Gamma(s) = \int_0^\infty \mathrm{d}t\, t^{s-1} e^{-t}.$$

All this means the logarithm of the notification likelihood with σ integrated out is

$$\log(M) = -\left[\alpha + (n+1)/2\right] \times \log(E+\beta) + \text{constant} \tag{29}$$

To deal with the fact that some notification data is stratified by age, we consider notifications in different age categories: $N_{t,a}$ where $a \in \mathbb{K} \cup \{\text{unknown}\}$ where $\mathbb{K} = \{0-4, 5-14, 15-24, 25-34, 35-44, 45-54, 55-64, 65+\}$. The category $N_{t,\text{unknown}}$ is made up as the difference between the total notifications $N_t = \sum_a N_{t,a}$ and those

with known age categories in each year, $N_{t,\text{known}}$. We assume no bias in which notifications are age stratified. We also want to model the error in each terms so they aggregate to the correct total

$$\operatorname{var}\left(N_{t,a} - \hat{N}_{t,a}\right) = v_{t,a}; \quad \sum_a v_{t,a} = v_t; \quad v_t = \sigma^2 F^2$$

We therefore take $v_{t,\text{unknown}} = v_t \times N_{t,\text{unknown}}/N_t$ and $v_{t,a} = v_t \times (N_{t,\text{known}}/N_t)/|\mathbb{K}|$ for $a \in \mathbb{K}$.

We can then use Equation 29 with

$$E = \sum_{t,a \in K} \frac{(N_{t,a} - f_{t,\text{known}} \times \hat{N}_{t,a})^2}{2 v_{t,a}} + \sum_t \frac{(N_{t,\text{unknown}} - f_{t,\text{unknown}} \times \hat{N}_t)^2}{2 v_{t,\text{unknown}}}$$

where $f_{t,\text{unknown}} = N_{t,\text{unknown}}/N_t$, $f_{t,\text{known}} = N_{t,\text{known}}/N_t$.

HIV

The log-likelihood component capturing patterns with respect to HIV used estimates of the prevalence of HIV within TB notifications to avoid double-counting information on the overall level of notifications. For countries reporting on HIV within TB notifications, we conducted a random-effects meta-analysis to estimate the mean (G) and variance (τ_G) of the logit-proportion of TB notifications with HIV. The log-likelihood was then

$$LL_h = -\frac{(G - \hat{G})^2}{2\tau_G^2},$$

where $\hat{G}$ is the model-predicted logit of the average proportion of TB notifications with HIV over the years with data.

1.5 Sensitivity analyses

We explored the impact of different assumptions by refitting and recomputing key results. The same methods were used to calibrate the model to data under these alternate assumptions, and to compute results. These results are reported in Section 2.5 in Figures 22, 23 and 24.

Proportion of TB that is extrapulmonary

The original choice was based on an analysis of notification data and matched the value used in the WHO estimation process (just under 20% EPTB). Unfortunately, routine data to inform the proportion of recorded TB that is EPTB are extremely variable: this variability likely reflects differences in coding and report practice as much as it does any underlying epidemiological variation, and by an unknown extent.

For our sensitivity analysis, we re-ran our whole analysis assuming either 30% or 10% for the fraction of TB that is EPTB. We calculated the incidence and mortality estimates for each country in 2019 (Figure 22) and also the impact of these assumptions on the proportion of TB incidence in 2019 due to (re)infection (Figure 23).

Infectiousness of 10-14 year olds

Our basecase assumption is that 10-14 year olds with TB are not infectious.

To inform an alternative assumption, we reviewed literature relevant to rates of bacteriological test positivity in children, since bacteriological test positivity is well-established to correlate with infectiousness. Kunkel et al's[19] systematic review and meta-analysis reported of smear-positivity in different age groups reported 0.5% (0-1.9) for 0-4 year olds; 14.0% (8.9-19.4) for 5-14 year olds; and 52% (40-64) for 15+ year olds. Unfortunately, the 10-14 year old category was not reported separately. Du Preez et al.[20] report on bacteriological confirmation among notified TB in South Africa 2004-2916 in 5 year age groups. They report bacteriological-confirmation rates of 3.1% in 0-4 year olds; 9.8% of 5-9 year olds; 37.2% of 10-14 year olds; and 59% of 15-19 year olds. Combining Du Preez et al's results across 5-9 year olds and 10-14 year olds gives 20% bacteriological confirmation in the 5-14 year old category for comparison with Kunkel et al.

The results of Du Preez et al suggest 63% as much bacteriological confirmation in 10-14 year olds as 15-19 year olds; comparison with Kunkel et al aggregated to matching age categories suggests perhaps a lower fraction of 10-14 year olds who are bacteriologically positive are smear-positive, consistent with expectations of a more paucibacillary spectrum of disease. Furthermore, it has been observed in other data (e.g. Vynnycky et al.[21]) that smear positivity rates show a slight gradient in adults, so that bacteriological positivity among 10-14 year olds may be lower compared with average adult TB than TB in 10-19 year olds.

Given this information, we refitted our model and generated results under the assumption that 10-14 year olds are 50% as infectious per unit time as adults. We made the assumption that the duration of untreated TB disease in this group was the same as adults with the same HIV/ART status, which is likely to be higher than reality, exaggerating the prevalence and importance of this group for transmission.

Unfortunately, there is scant data to quantify disease duration in children. There is very little data on the prevalence of TB in children <15 years in the general popu-

lation, as children are routinely excluded from TB prevalence surveys on grounds of practicality, expense, and ethics. Data from household contacts (Martinez et al[11]) support a more rapid disease in young children than adults, but are indirect and have fewer older children. For this reason, we have not reported on the prevalence of TB in children - it lacks empirical data to compare against, and is not relevant for transmission in the basecase analysis. Our default assumption of 6 months (which we also use in this sensitivity analysis) only means that mortality in children is less lagged with respect to incidence than in adults.

In addition to the effect of this assumption on incidence, mortality and the proportion of TB incidence due to 'recent' transmission (Figures 22 and 23), we also calculated the proportion of all TB transmission in 2019 coming from 10-14 year olds (Figure 24).

2 Supplementary Results

2.1 AIM HIV fits: red lines are simplified AIM model fits from pursuit algorithm; black points are data associated with the full AIM model

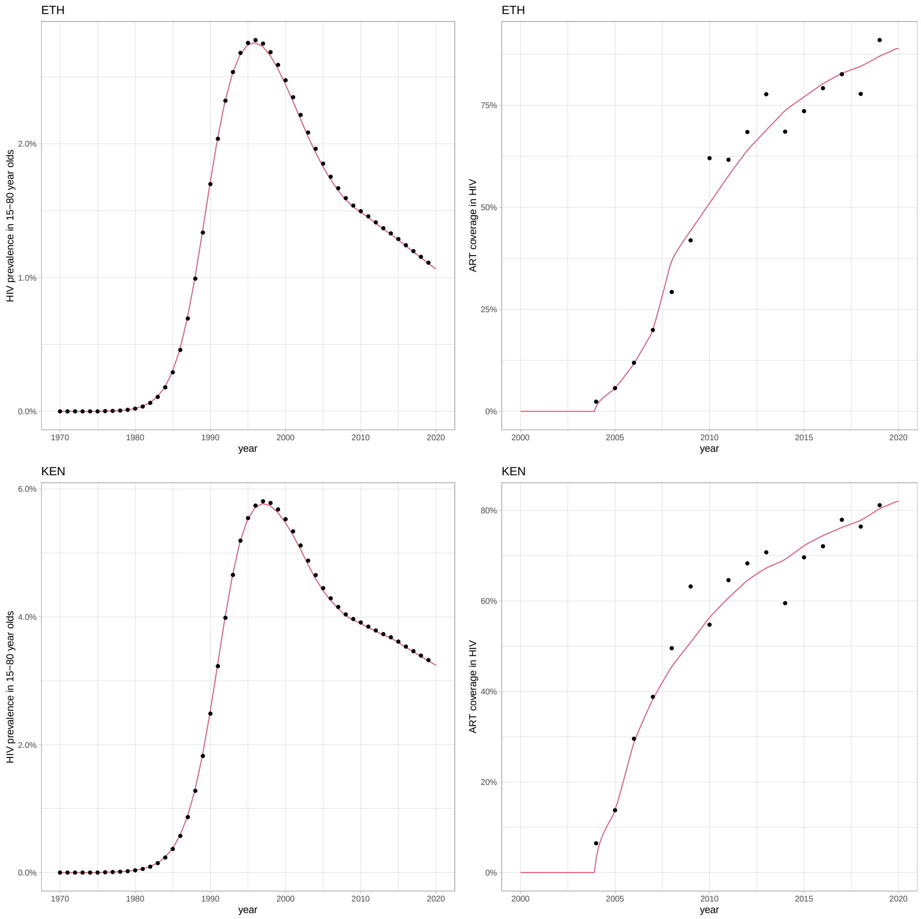

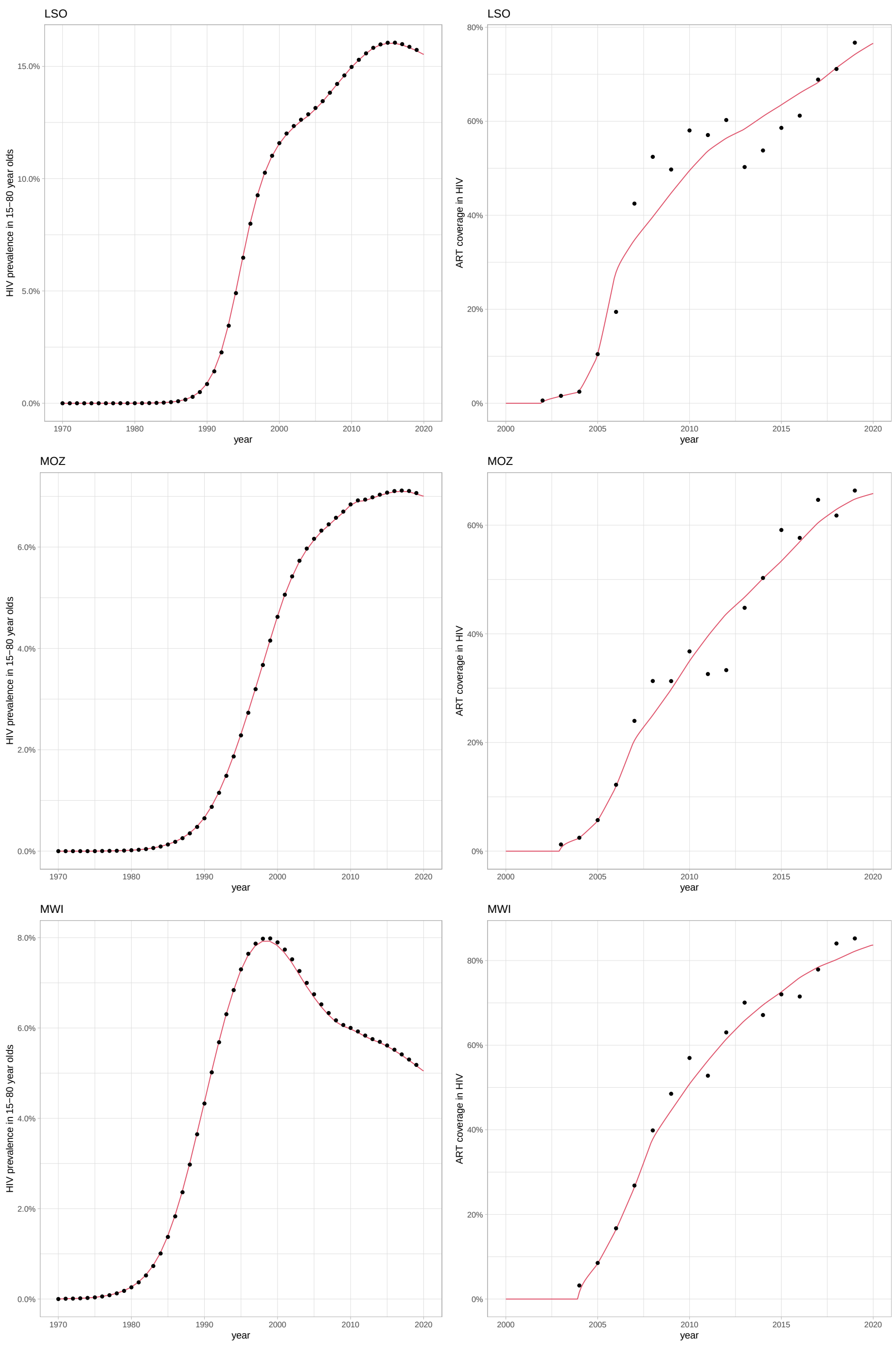

LSO
HIV prevalence in 15–80 year olds
0.0%
5.0%
10.0%
15.0%
1970
1980
1990
2000
2010
2020
year
LSO
ART coverage in HIV
0%
20%
40%
60%
80%
2000
2005
2010
2015
2020
year
MOZ
HIV prevalence in 15–80 year olds
0.0%
2.0%
4.0%
6.0%
MOZ
ART coverage in HIV
MWI
HIV prevalence in 15–80 year olds
8.0%
MWI
ART coverage in HIV

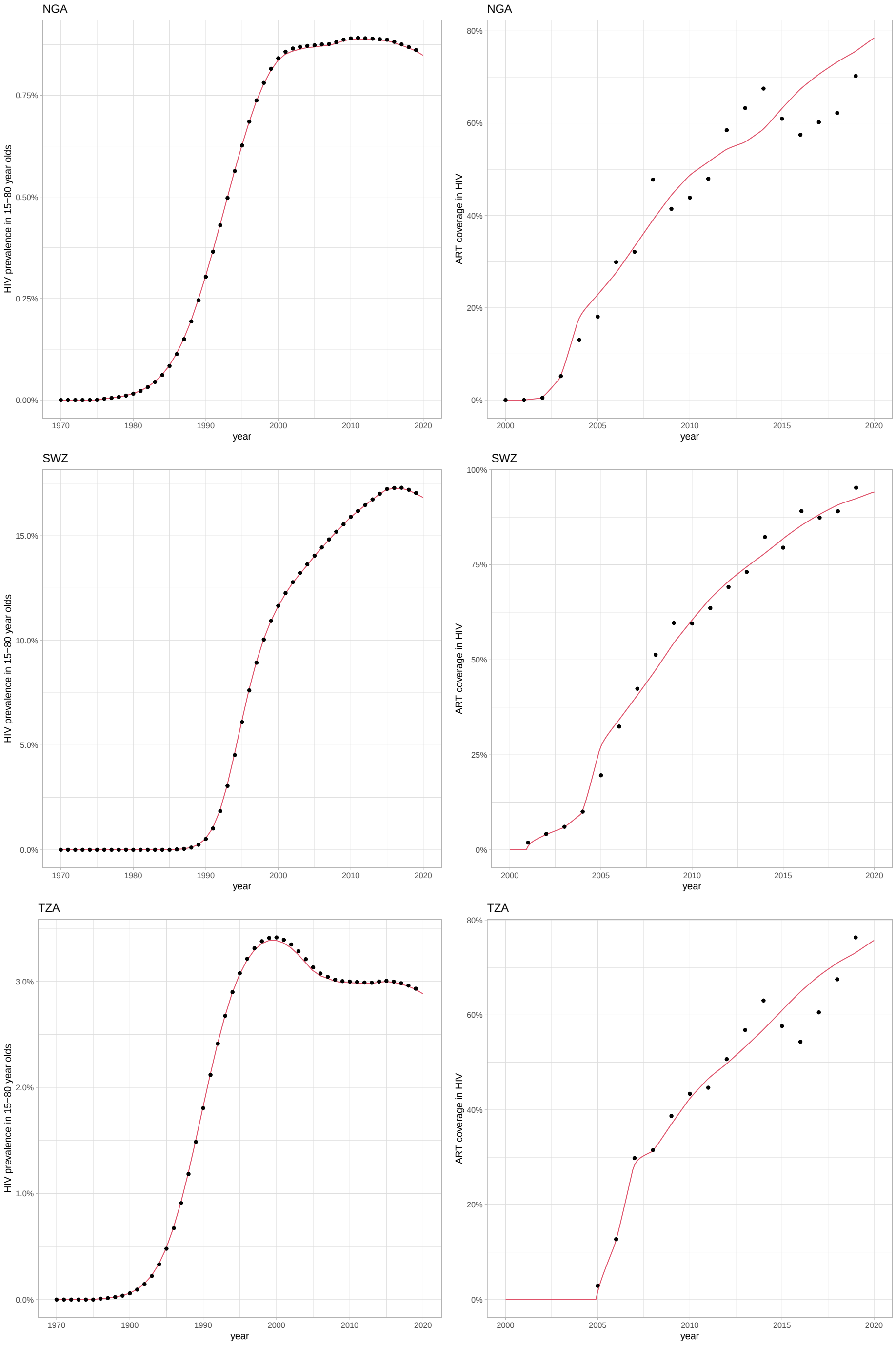
NGA
HIV prevalence in 15–80 year olds
0.00%
0.25%
0.50%
0.75%
1970
1980
1990
2000
2010
2020
year
NGA
ART coverage in HIV
0%
20%
40%
60%
80%
2000
2005
2010
2015
2020
year
SWZ
HIV prevalence in 15–80 year olds
0.0%
5.0%
10.0%
15.0%
1970
1980
1990
2000
2010
2020
year
SWZ
ART coverage in HIV
0%
25%
50%
75%
100%
2000
2005
2010
2015
2020
year
TZA
HIV prevalence in 15–80 year olds
0.0%
1.0%
2.0%
3.0%
1970
1980
1990
2000
2010
2020
year
TZA
ART coverage in HIV
0%
20%
40%
60%
80%
2000
2005
2010
2015
2020
year

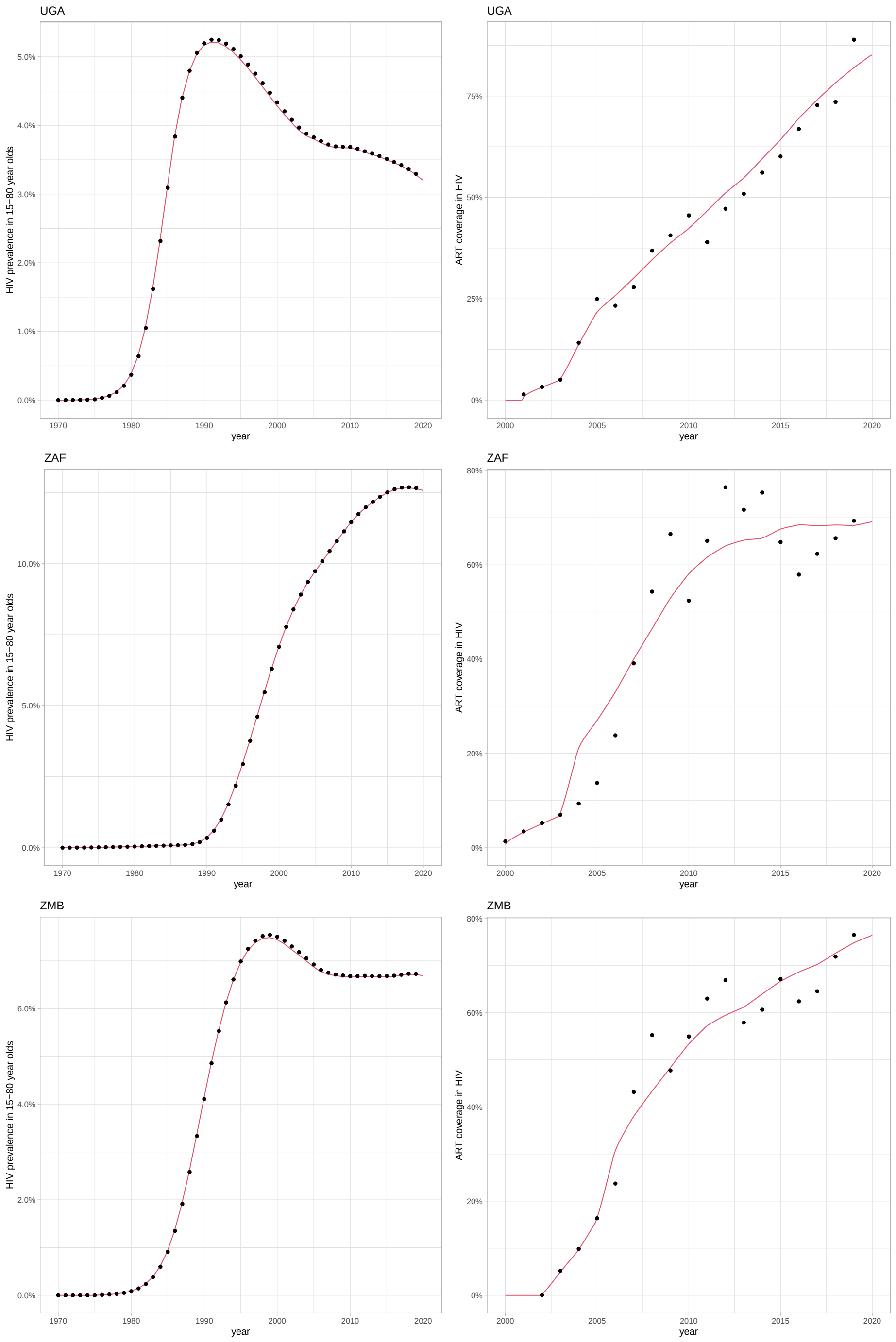
UGA
HIV prevalence in 15−80 year olds
0.0%
1.0%
2.0%
3.0%
4.0%
5.0%
1970
1980
1990
2000
2010
2020
year
UGA
ART coverage in HIV
0%
25%
50%
75%
2000
2005
2010
2015
2020
year
ZAF
HIV prevalence in 15−80 year olds
0.0%
5.0%
10.0%
1970
1980
1990
2000
2010
2020
year
ZAF
ART coverage in HIV
0%
20%
40%
60%
80%
2000
2005
2010
2015
2020
year
ZMB
HIV prevalence in 15−80 year olds
0.0%
2.0%
4.0%
6.0%
1970
1980
1990
2000
2010
2020
year
ZMB
ART coverage in HIV
0%
20%
40%
60%
80%
2000
2005
2010
2015
2020
year

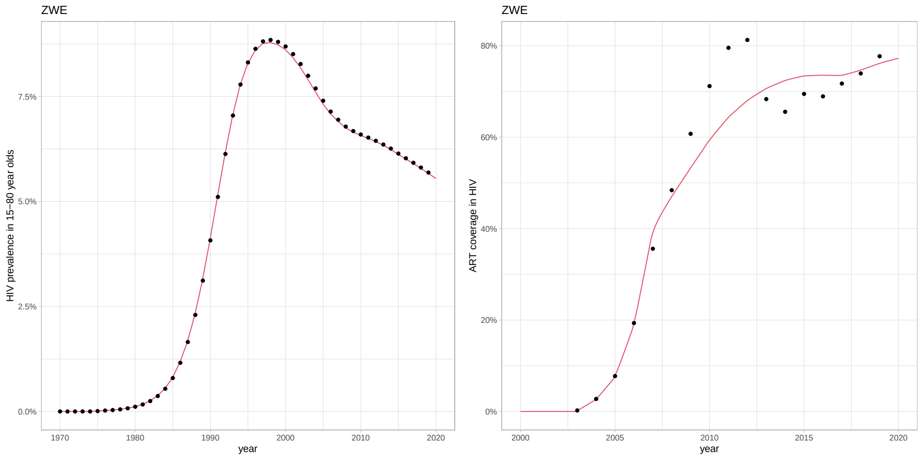

ZWE
HIV prevalence in 15–80 year olds
0.0%
2.5%
5.0%
7.5%
1970
1980
1990
2000
2010
2020
year
ZWE
ART coverage in HIV
0%
20%
40%
60%
80%
2000
2005
2010
2015
2020
year

2.2 Inference outputs

Figure 5 allows an assessment of which parameters have been most informed by the data under inference, i.e. which have shifted most in order to achieve fit, and whether these are similar across different countries. For the parameters that are weakly informed by data, this figure provides a check that the uncertainty has been thoroughly sampled (e.g. the case fatality parameters in the bottom row).

The corner plots in Section 2.3 show bivariate posteriors and give a sense of the adequacy of inference in having sufficient sample size to produce smooth 2D density plots (and the easier 1D density plots) as well as the capacity to detect meaningful correlations between parameters. E.g. the detection level parameter (K) and the detection rate of change parameter (c) are often negatively correlated, indicating high values of c can compensate for low initial detection in achieving fits. Similarly, the transmission parameter (β) and the slow progression parameter (ν) are often negatively correlated, indicating that higher transmission can compensate for lower progression in achieving fits.

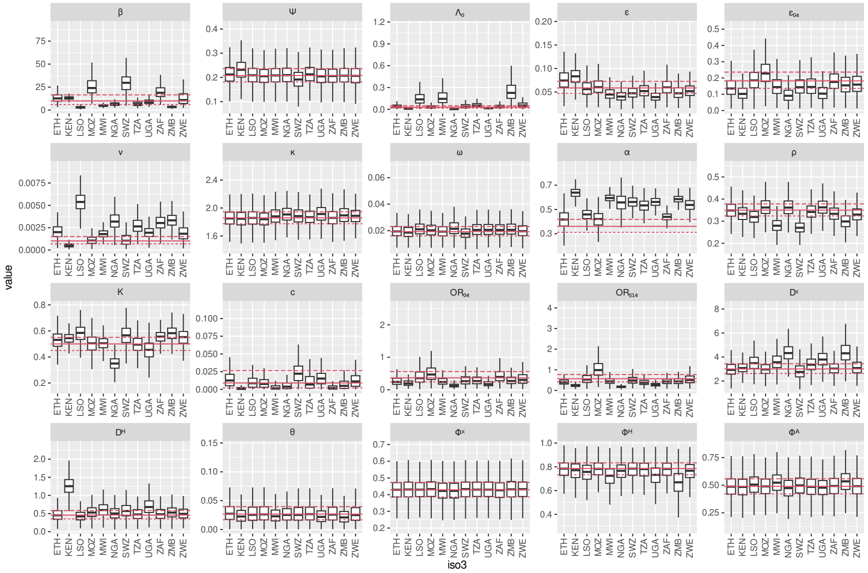

Figure 5: Posteriors vs priors. Red lines show prior medians (solid lines) and upper/lower quartiles (dashed lines) for each model parameter (panels). The box and whisker plots show posterior medians (mid-lines) and upper/lower quartiles (box upper/lower edges) as inferred for each country (ISO3 code on x-axis). Whiskers extend to the largest/smallest data point within 1.5× interquartile range of the median. This shows which parameters adjust most to achieve fit and that non-influential parameters have their uncertainty fully represented. Based on n=50,000 Markov chain Monte Carlo samples from the posterior.

2.3 Corner plots

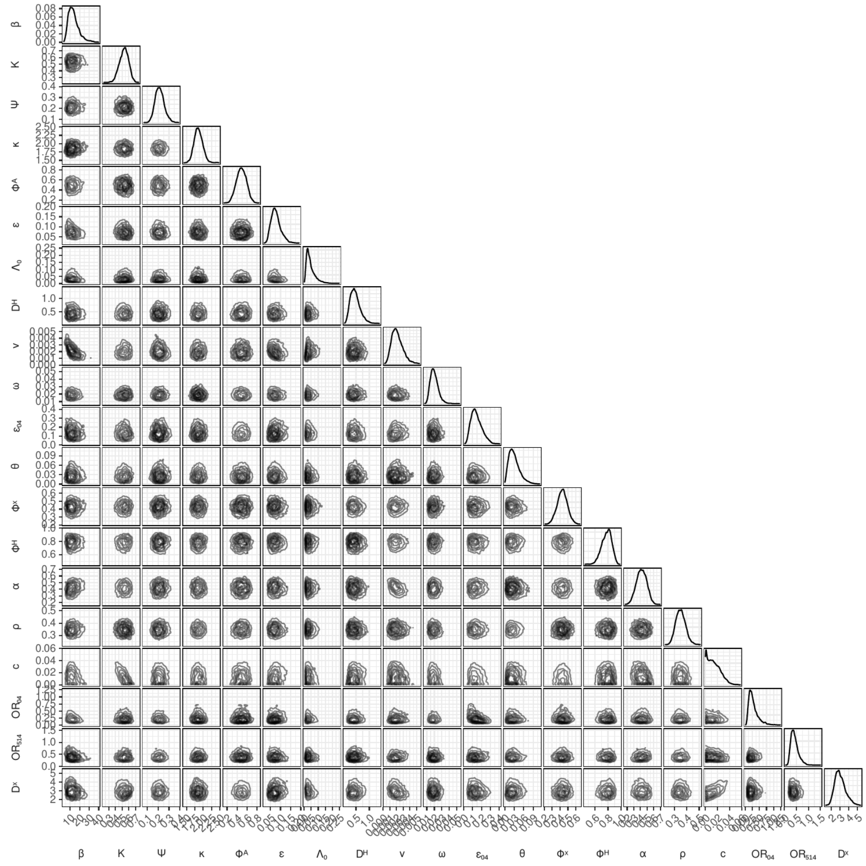

Figure 6: Corner plot for ETH showing bivariate posteriors and marginals.

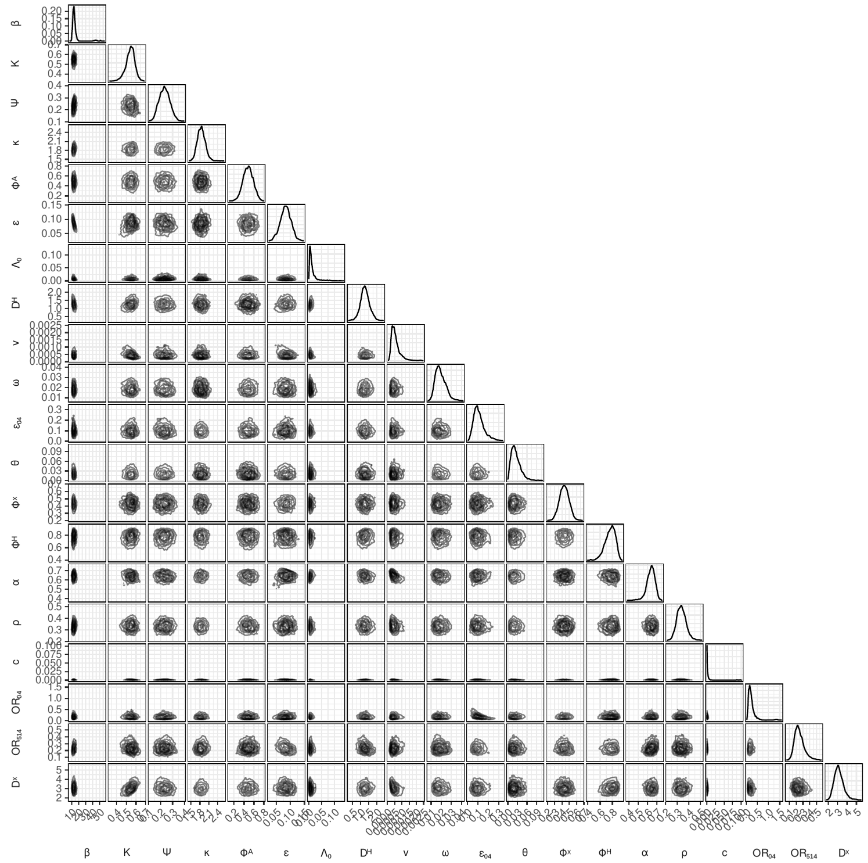

Figure 7: Corner plot for KEN showing bivariate posteriors and marginals.

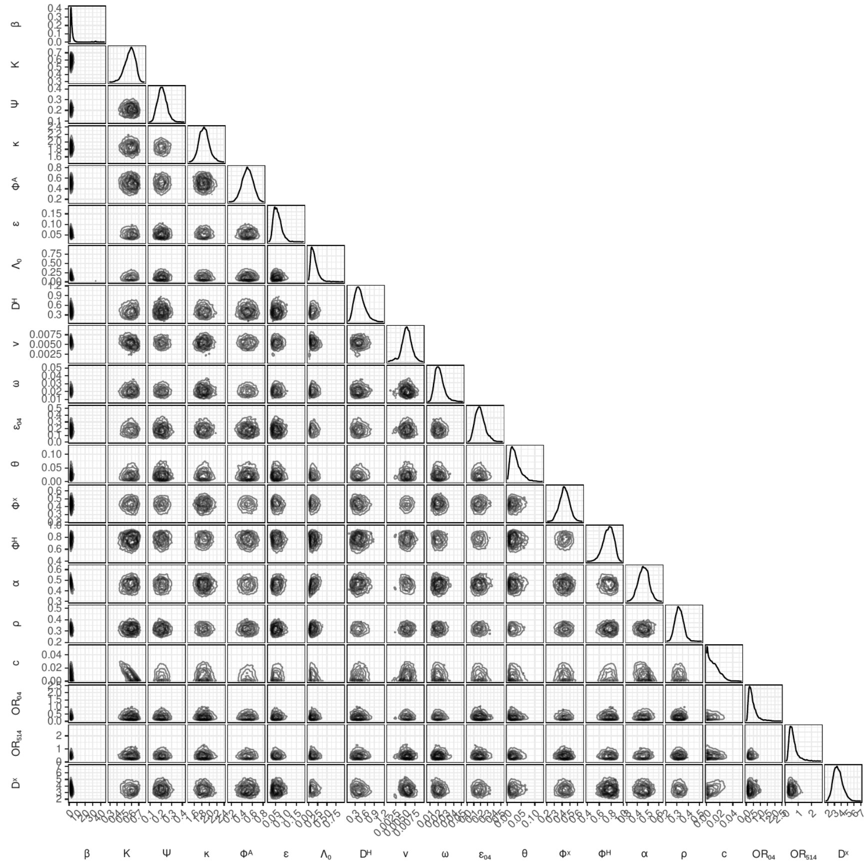

Figure 8: Corner plot for LSO showing bivariate posteriors and marginals.

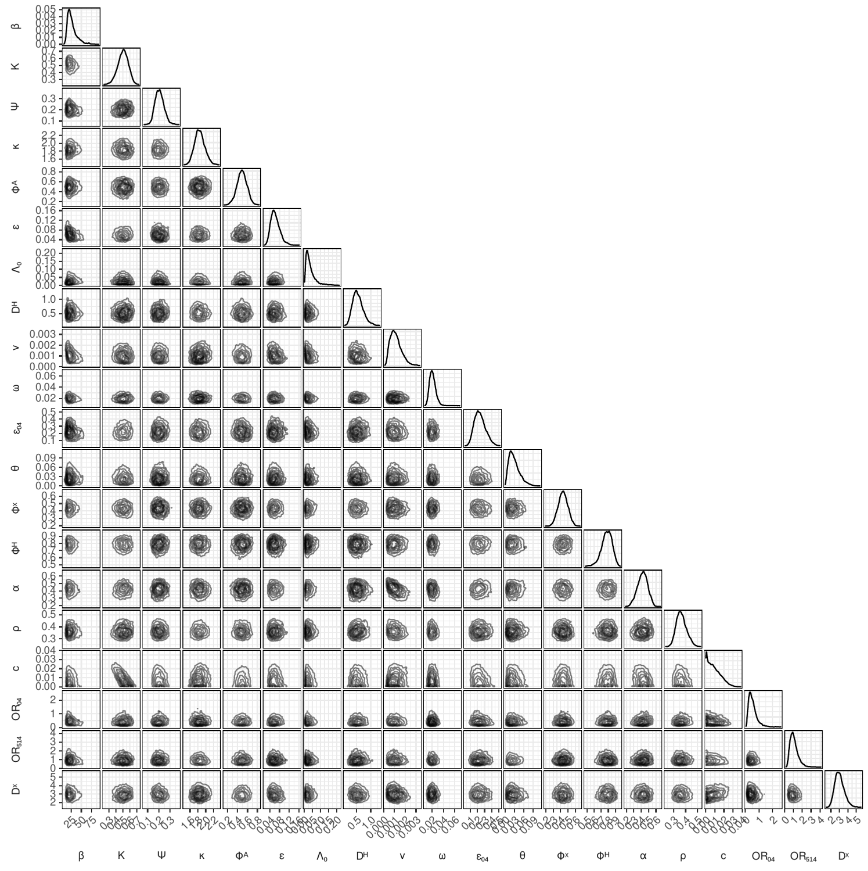

Figure 9: Corner plot for MOZ showing bivariate posteriors and marginals.

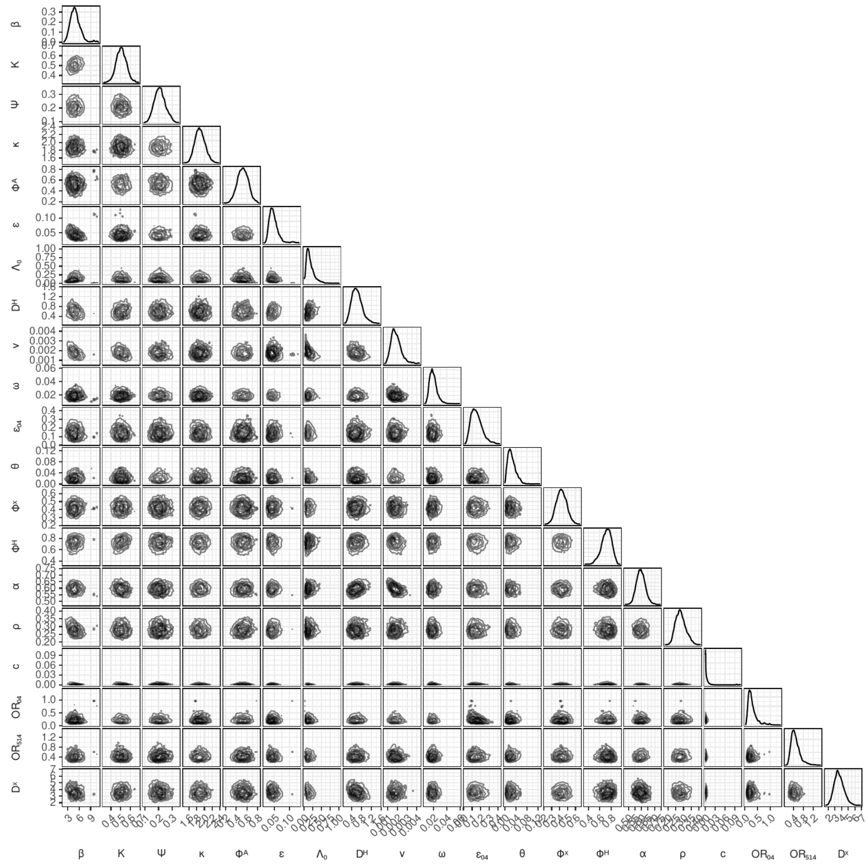

Figure 10: Corner plot for MWI showing bivariate posteriors and marginals.

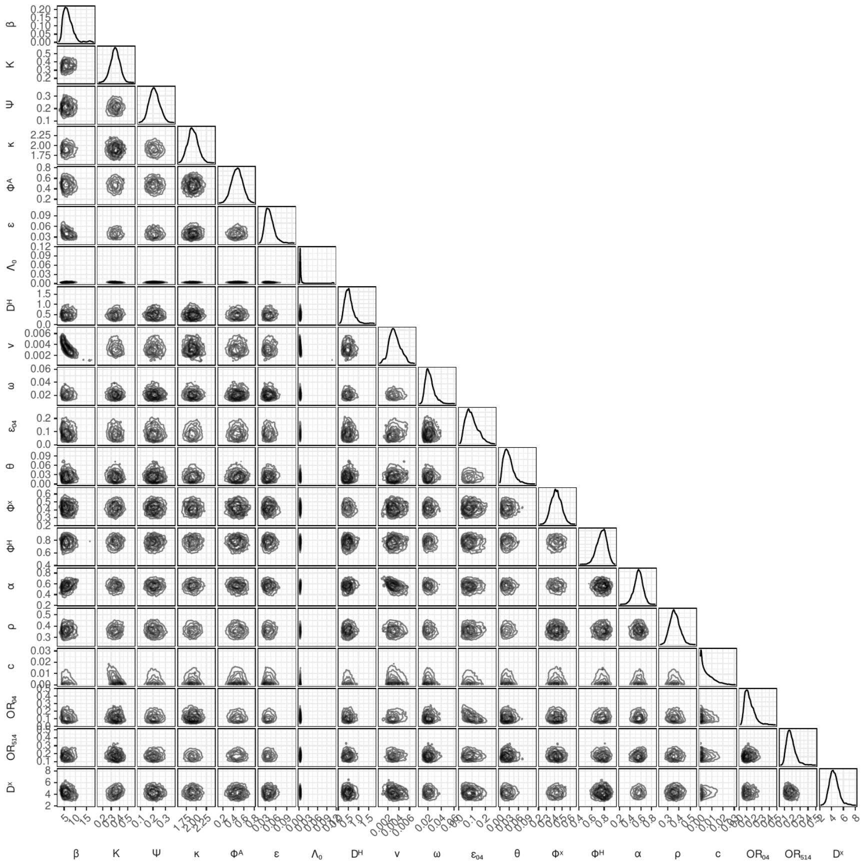

Figure 11: Corner plot for NGA showing bivariate posteriors and marginals.

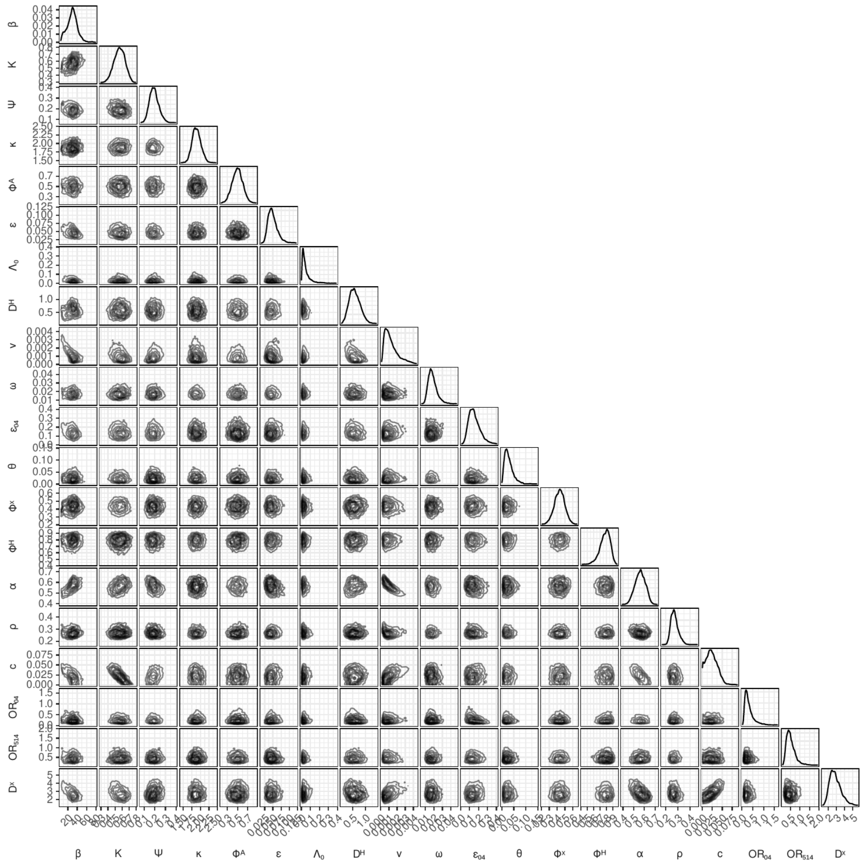

Figure 12: Corner plot for SWZ showing bivariate posteriors and marginals.

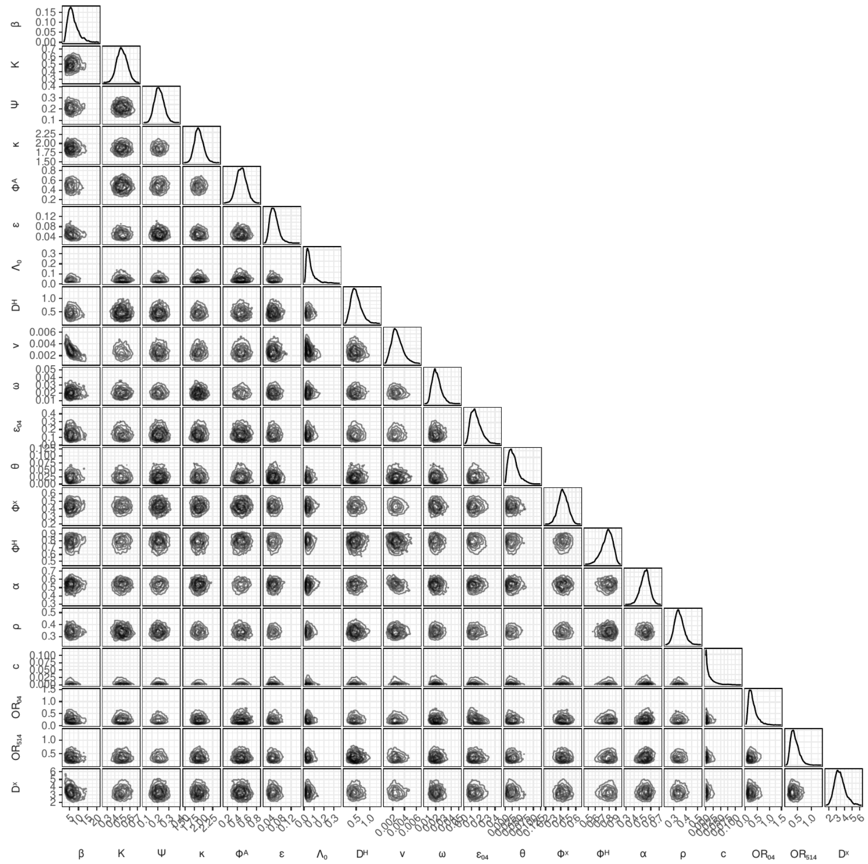

Figure 13: Corner plot for TZA showing bivariate posteriors and marginals.

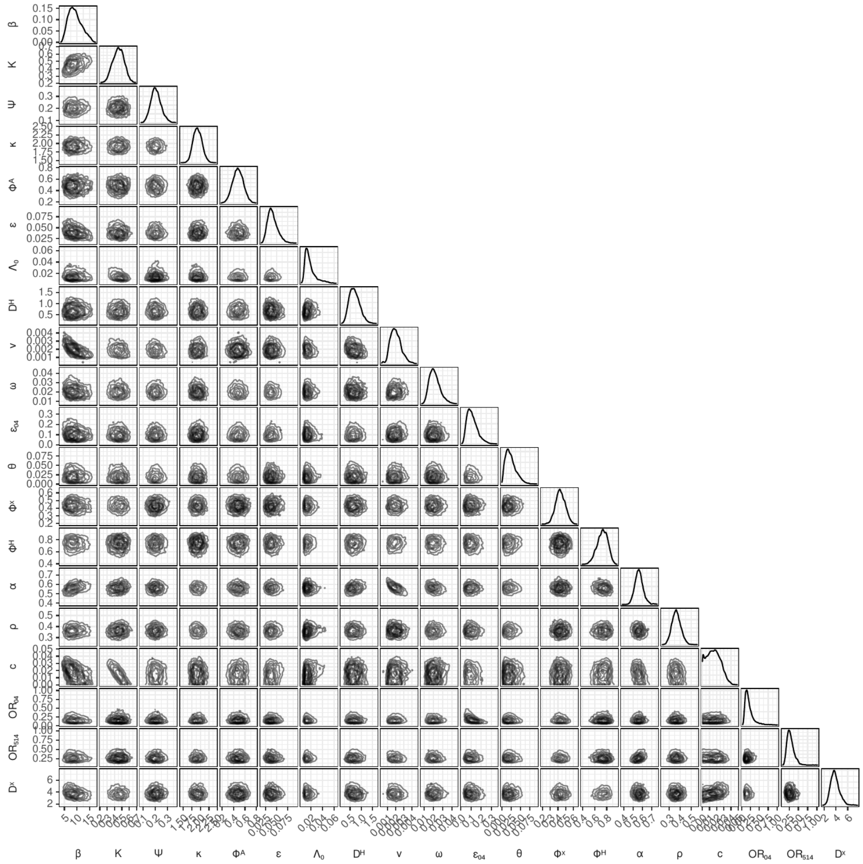

Figure 14: Corner plot for UGA showing bivariate posteriors and marginals.

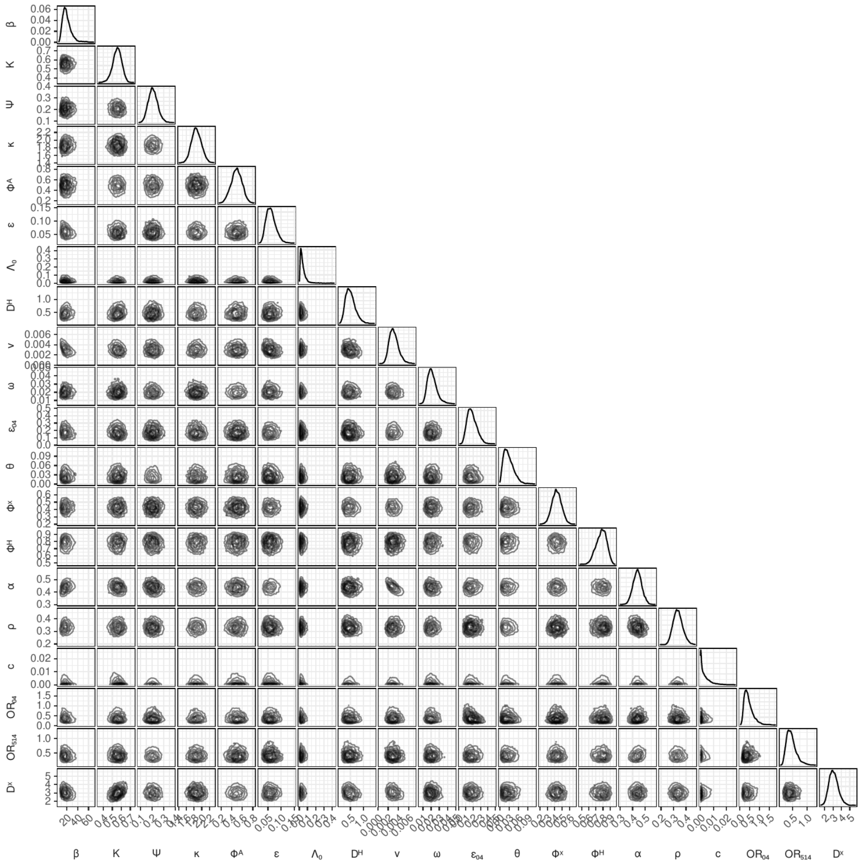

Figure 15: Corner plot for ZAF showing bivariate posteriors and marginals.

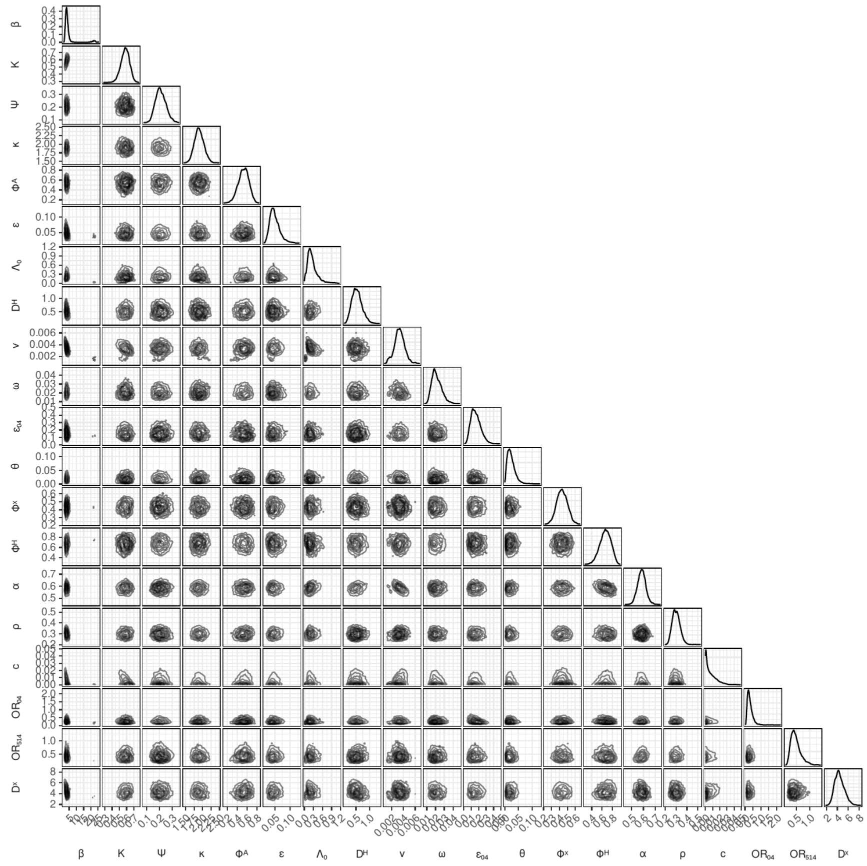

Figure 16: Corner plot for ZMB showing bivariate posteriors and marginals.

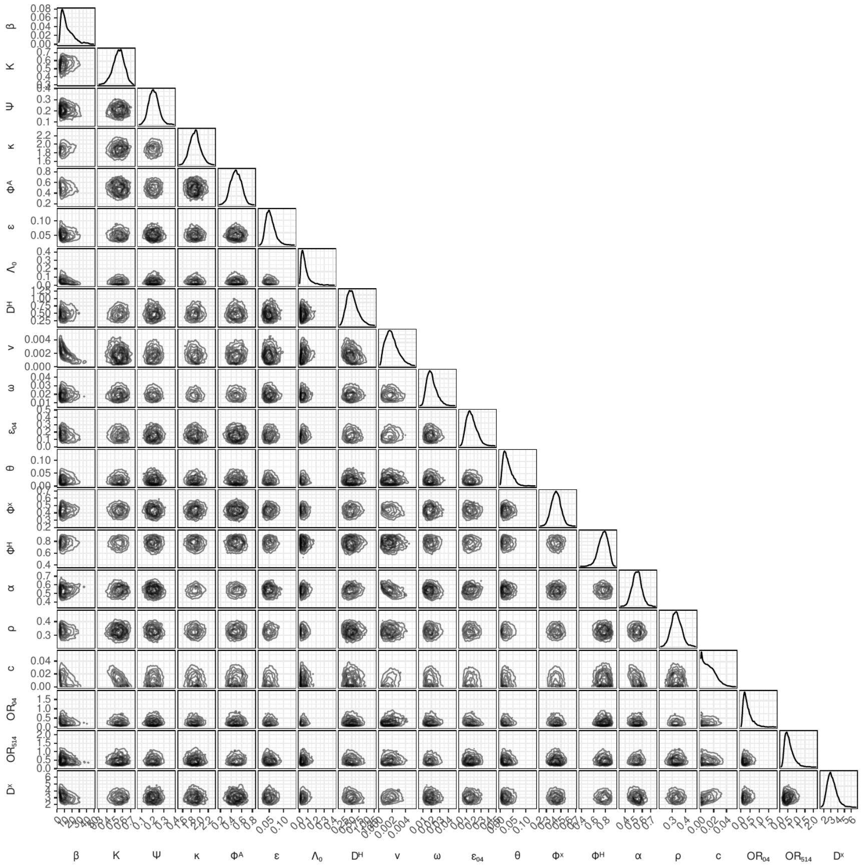

Figure 17: Corner plot for ZWE showing bivariate posteriors and marginals.

2.4 Epidemiological plots with uncertainty

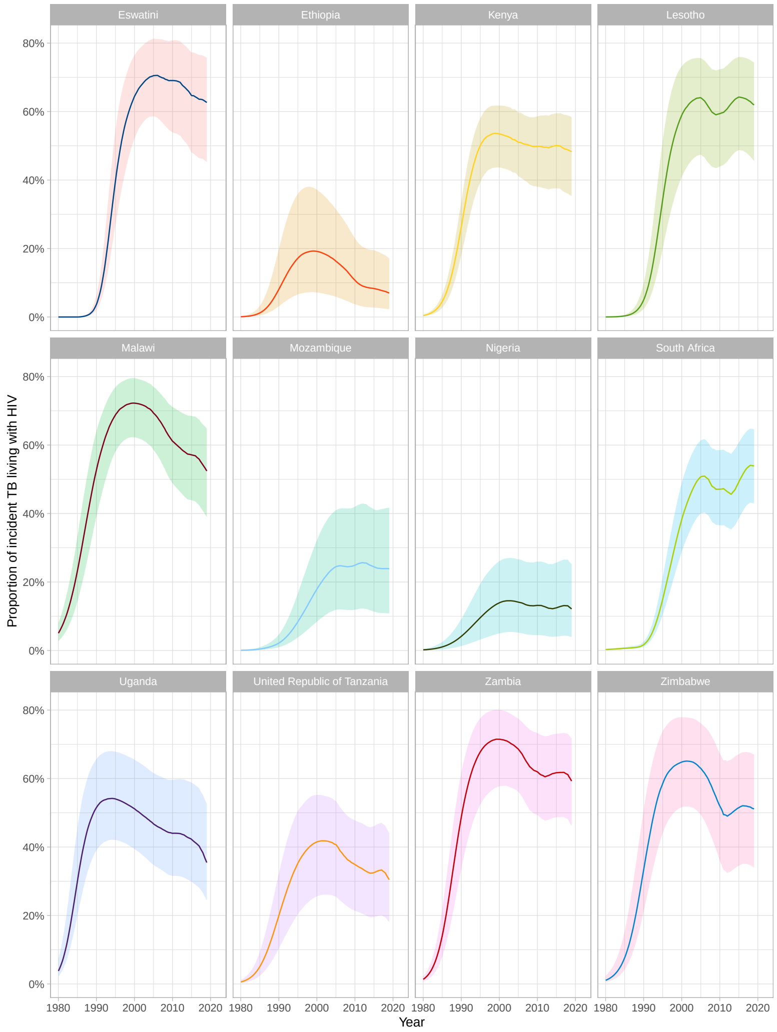

Figure 18: The proportion of incident TB that is TB/HIV for 1980-2019, ribbon=95%CrI, line=median.

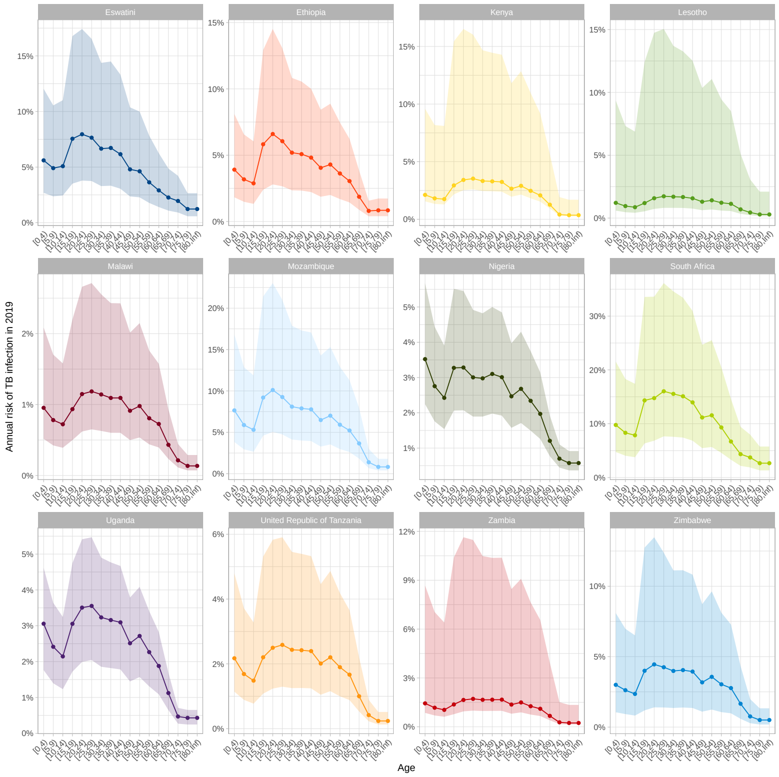

Figure 19: Annual risk of TB infection in 2019 by age, ribbon=95%CrI, point/line=median.

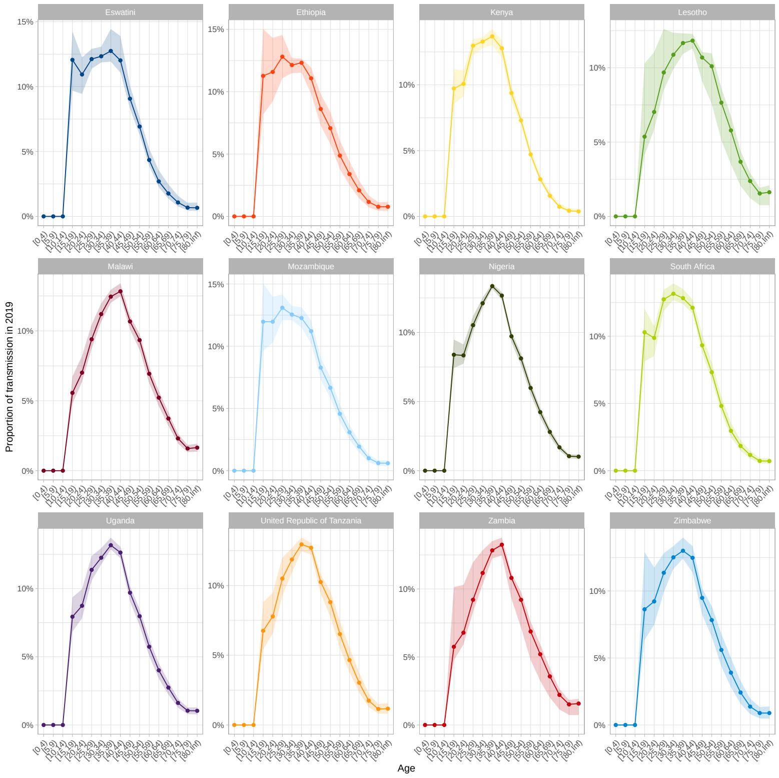

Figure 20: Proportion of TB transmission from each age group in 2019, ribbon=95%CrI, point/line=median.

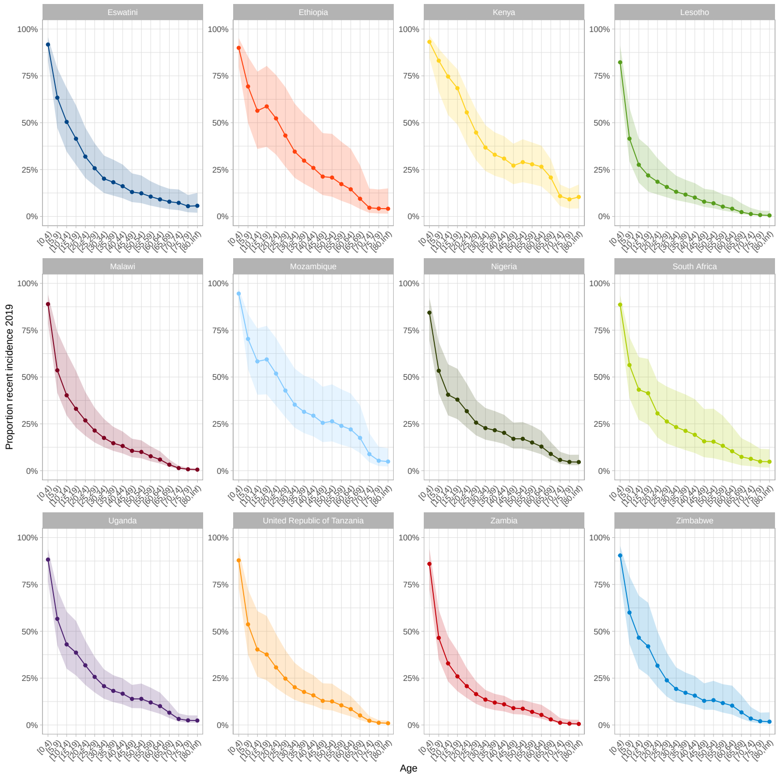

Figure 21: Proportion of all TB incidence in 2019 due to (re)infection within 2 years for each age group, ribbon=95%CrI, point/line=median.

2.5 Sensitivity analysis results

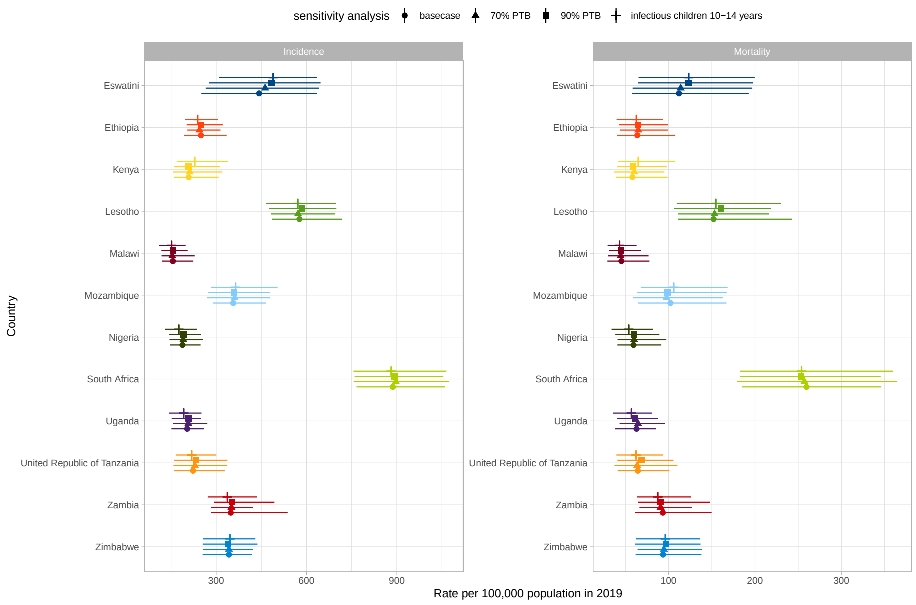

Figure 22: Impact of sensitivity analyses on calibrated estimates of TB incidence and mortality in 2019. Points show medians and uncertainty intervals show the 2.5% and 97.5% quantiles based on runs using n=300 random samples from the posterior.

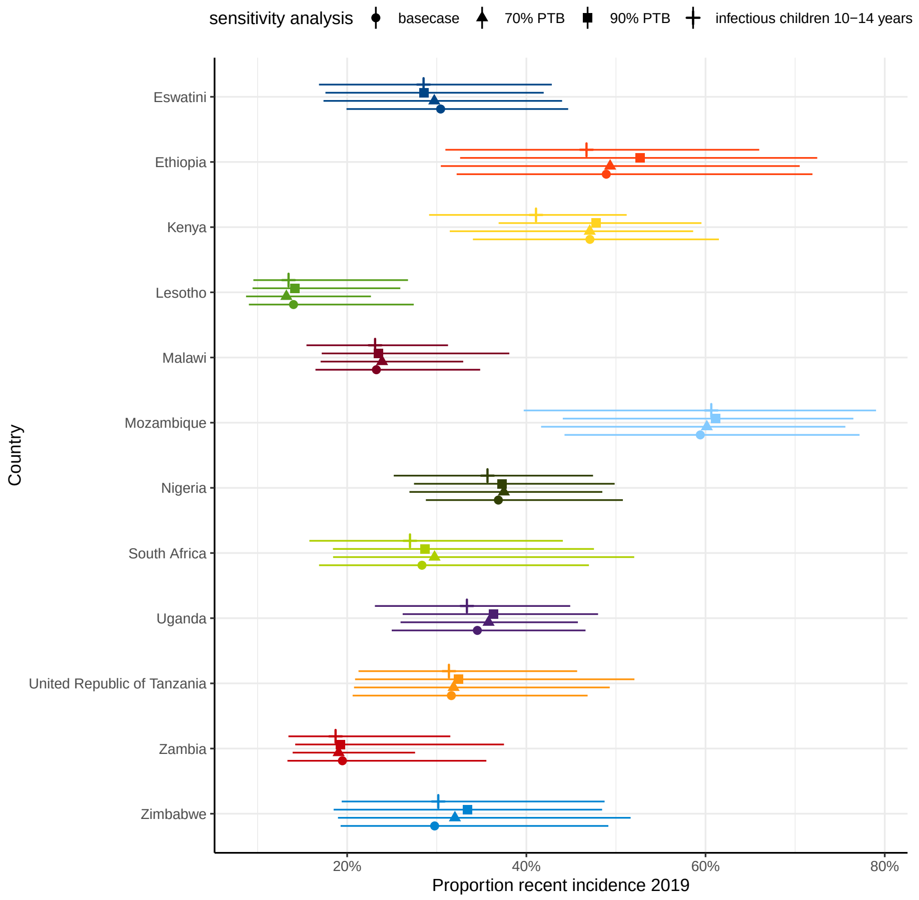

Figure 23: Impact of sensitivity analyses on estimates of the proportion of TB incidence in 2019 due to (re)infection within 2 years. Points show medians and uncertainty intervals show the 2.5% and 97.5% quantiles based on runs using n=300 random samples from the posterior.

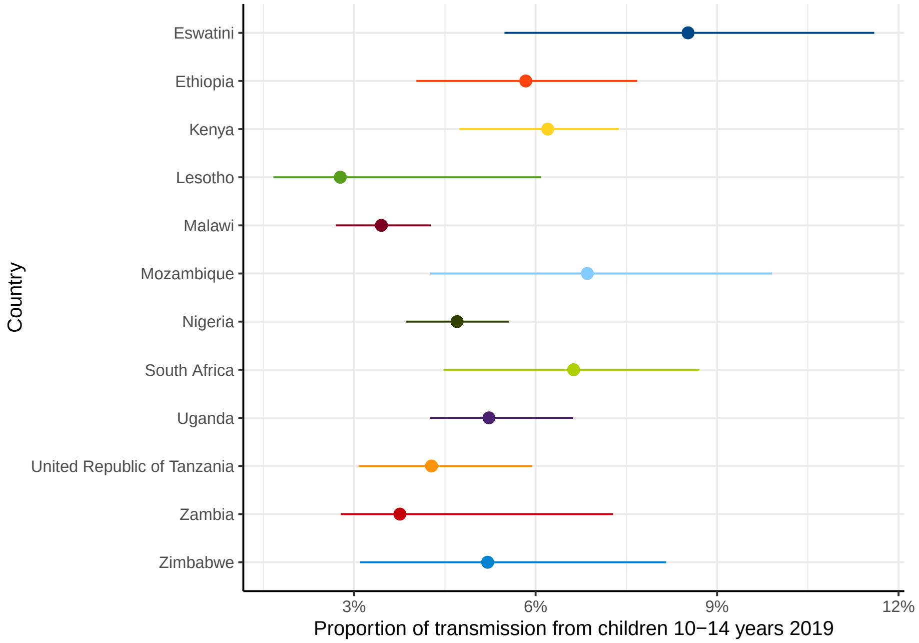

Figure 24: Proportion of all TB transmission from 10-14 year olds when assuming they are 50% as infectious as adults. Points show medians and uncertainty intervals show the 2.5% and 97.5% quantiles based on runs using n=300 random samples from the posterior.

References

[1] Joint United Nations Programme on HIV/ AIDS. IN DANGER: UNAIDS global AIDS update 2022. Technical report, 2022.

[2] R Core Team. *R: A Language and Environment for Statistical Computing*. R Foundation for Statistical Computing, Vienna, Austria, 2022.

[3] Penelope K Ellis, Willam J Martin, and Peter J Dodd. CD4 count and tuberculosis risk in HIV-positive adults not on ART: a systematic review and meta-analysis. *PeerJ*, 5(e4165):e4165, December 2017.

[4] Brian G Williams, Reuben Granich, Kevin M De Cock, Philippe Glaziou, Abhishek Sharma, and Christopher Dye. Antiretroviral therapy for tuberculosis control in nine african countries. *Proc. Natl. Acad. Sci. U. S. A.*, 107(45):19485–19489, November 2010.

[5] Luuk Gras, Anouk M Kesselring, James T Griffin, Ard I van Sighem, Christophe Fraser, Azra C Ghani, Frank Miedema, Peter Reiss, Joep M A Lange, Frank de Wolf, and ATHENA, Netherlands National Observational Cohort Study. CD4 cell counts of 800 cells/mm3 or greater after 7 years of highly active antiretroviral therapy are feasible in most patients starting with 350 cells/mm3 or greater. *J. Acquir. Immune Defic. Syndr.*, 45(2):183–192, June 2007.

[6] Denis Nash, Monica Katyal, Martin W G Brinkhof, Olivia Keiser, Margaret May, Rachael Hughes, Francois Dabis, Robin Wood, Eduardo Sprinz, Mauro Schechter, Matthias Egger, and ART-LINC Collaboration of IeDEA. Long-term immunologic response to antiretroviral therapy in low-income countries: a collaborative analysis of prospective studies. *AIDS*, 22(17):2291–2302, November 2008.

[7] Rich FitzJohn and Travis Fischer. *odin: ODE Generation and Integration*, 2022. R package version 1.3.6.

[8] Karline Soetaert, Thomas Petzoldt, and R. Woodrow Setzer. Solving differential equations in R: Package deSolve. *Journal of Statistical Software*, 33(9):1–25, 2010.

[9] Kiesha Prem, Kevin van Zandvoort, Petra Klepac, Rosalind M Eggo, Nicholas G Davies, Centre for the Mathematical Modelling of Infectious Diseases COVID-19 Working Group, Alex R Cook, and Mark Jit. Projecting contact matrices in 177 geographical regions: An update and comparison with empirical data for the COVID-19 era. *PLoS Comput. Biol.*, 17(7):e1009098, July 2021.

[10] Romain Ragonnet, James M Trauer, Nick Scott, Michael T Meehan, Justin T Denholm, and Emma S McBryde. Optimally capturing latency dynamics in models of tuberculosis transmission. *Epidemics*, 21:39–47, June 2017.

[11] Leonardo Martinez, Olivia Cords, C Robert Horsburgh, Jason R Andrews, Carlos Acuna-Villaorduna, Shama Desai Ahuja, Neus Altet, Orvalho Augusto, Davit Baliashvili, Sanjay Basu, Mercedes Becerra, Maryline Bonnet, W Henry Boom, Martien Borgdorff, Fadila Boulahbal, Anna Cristina C Carvalho, Joan A Cayla, Tsira Chakhaia, Pei-Chun Chan, Ted Cohen, Julio Croda, Sumona Datta, Helena del Corral, Justin T Denholm, Reynaldo Dietze, Claudia C Dobler, Simon Donkor, Uzochukwu Egere, Jerrold J Ellner, Marcos Espinal, Carlton A Evans, Chi-Tai Fang, Katherine Fielding, Greg J Fox, Luis F García, Alberto L García-Basteiro, Steffen Geis, Stephen M Graham, Louis Grandjean, Djohar Hannoun, Mark Hatherill, Anja M Hauri, Anneke C Hesseling, Philip C Hill, Li-Min Huang, Helena Huerga, Rabia Hussain, Leah Jarlsberg, Edward C Jones-López, Seiya Kato, Midori Kato-Maeda, Beate Kampmann, H Lester Kirchner, Afrânio Kritski, Christoph Lange, Chih-Hsin Lee, Li-Na Lee, Meng-Rui Lee, Antonio Carlos Lemos, Christian Lienhardt, Du-Lin Ling, Qiao Liu, Nathan C Lo, Richard Long, Elisa Lopez-Varela, Peng Lu, Matthew Magee, Lashaunda L Malone, Anna M Mandalakas, Neil A Martinson, Rufaida Mazahir, Megan B Murray, Eduardo Martins Netto, Larissa Otero, Julie Parsonnet, Arthur Reingold, H Simon Schaaf, James A Seddon, Surendra Sharma, Jitendra Singh, Sarman Singh, Rosa Sloot, Giovanni Sotgiu, Catherine M Stein, Najeeha Talat Iqbal, Rina Triasih, Lisa Trieu, Maarten F Schim van der Loeff, Patrick Van der Stuyft, Cari van Schalkwyk, Richa Vashishtha, Lilly M Verhagen, Julian A Villalba, Jann-Yuan Wang, Christopher C Whalen, Takashi Yoshiyama, Heather J Zar, Jean-Pierre Zellweger, and Limei Zhu. The risk of tuberculosis in children after close exposure: a systematic review and individual-participant meta-analysis. *Lancet*, 395(10228):973–984, March 2020.

[12] Edine W Tiemersma, Marieke J van der Werf, Martien W Borgdorff, Brian G Williams, and Nico J D Nagelkerke. Natural history of tuberculosis: duration and fatality of untreated pulmonary tuberculosis in HIV negative patients: a systematic review. *PLoS One*, 6(4):e17601, April 2011.

[13] P Glaziou, P J Dodd, A Dean, and K Floyd. Methods used by WHO to estimate the global burden of TB disease. 2020.

[14] Peter J Dodd, Elizabeth Gardiner, Renia Coghlan, and James A Seddon. Burden of childhood tuberculosis in 22 high-burden countries: a mathematical modelling study. *Lancet Glob Health*, 2(8):e453–9, August 2014.

[15] Jason R Andrews, Farzad Noubary, Rochelle P Walensky, Rodrigo Cerda, Elena Losina, and C Robert Horsburgh. Risk of progression to active tuberculosis following reinfection with *Mycobacterium tuberculosis*. *Clin. Infect. Dis.*, 54(6):784–791, March 2012.

[16] Amelia C Crampin, J Nimrod Mwaungulu, Frank D Mwaungulu, D Totah Mwafulirwa, Kondwani Munthali, Sian Floyd, Paul Em Fine, and Judith R Glynn. Recurrent TB: relapse or reinfection? The effect of HIV in a general population cohort in Malawi. *AIDS*, 24(3):417–426, January 2010.

[17] Amitabh B Suthar, Stephen D Lawn, Julia del Amo, Haileyesus Getahun, Christopher Dye, Delphine Sculier, Timothy R Sterling, Richard E Chaisson, Brian G Williams, Anthony D Harries, and Reuben M Granich. Antiretroviral therapy for prevention of tuberculosis in adults with HIV: a systematic review and meta-analysis. *PLoS Med.*, 9(7):e1001270, July 2012.

[18] Chu-Chang Ku, Peter MacPherson, Mcewen Khundi, Rebecca H Nzawa Soko, Helena R A Feasey, Marriott Nliwasa, Katherine C Horton, Elizabeth L Corbett, and Peter J Dodd. Durations of asymptomatic, symptomatic, and care-seeking phases of tuberculosis disease with a bayesian analysis of prevalence survey and notification data. *BMC Med.*, 19(1):1–13, November 2021.

[19] Amber Kunkel, Pia Abel Zur Wiesch, Ruvandhi R Nathavitharana, Florian M Marx, Helen E Jenkins, and Ted Cohen. Smear positivity in paediatric and adult tuberculosis: systematic review and meta-analysis. *BMC Infect. Dis.*, 16:282, June 2016.

[20] Karen du Preez, Muhammad Osman, James A Seddon, Pren Naidoo, H Simon Schaaf, Zahn Munch, Rory Dunbar, Lindiwe Mvusi, Sicelo S Dlamini, and Anneke C Hesseling. The impact of the evolving HIV response on the epidemiology of tuberculosis in south african children and adolescents. *Clin. Infect. Dis.*, February 2021.

[21] E Vynnycky and P E Fine. The natural history of tuberculosis: the implications of age-dependent risks of disease and the role of reinfection. *Epidemiol. Infect.*, 119(2):183–201., October 1997.
